# Subcellular Localization Determines the Stability and Axon Protective Capacity of Axon Survival Factor Nmnat2

**DOI:** 10.1371/journal.pbio.1001539

**Published:** 2013-04-16

**Authors:** Stefan Milde, Jonathan Gilley, Michael P. Coleman

**Affiliations:** The Babraham Institute, Cambridge, United Kingdom; Stanford University School of Medicine, United States of America

## Abstract

Axons require a constant supply of the labile axon survival factor Nmnat2 from their cell bodies to avoid spontaneous axon degeneration. Here we investigate the mechanism of fast axonal transport of Nmnat2 and its site of action for axon maintenance. Using dual-colour live-cell imaging of axonal transport in SCG primary culture neurons, we find that Nmnat2 is bidirectionally trafficked in axons together with markers of the trans-Golgi network and synaptic vesicles. In contrast, there is little co-migration with mitochondria, lysosomes, and active zone precursor vesicles. Residues encoded by the small, centrally located exon 6 are necessary and sufficient for stable membrane association and vesicular axonal transport of Nmnat2. Within this sequence, a double cysteine palmitoylation motif shared with GAP43 and surrounding basic residues are all required for efficient palmitoylation and stable association with axonal transport vesicles. Interestingly, however, disrupting this membrane association increases the ability of axonally localized Nmnat2 to preserve transected neurites in primary culture, while re-targeting the strongly protective cytosolic mutants back to membranes abolishes this increase. Larger deletions within the central domain including exon 6 further enhance Nmnat2 axon protective capacity to levels that exceed that of the slow Wallerian degeneration protein, Wld^S^. The mechanism underlying the increase in axon protection appears to involve an increased half-life of the cytosolic forms, suggesting a role for palmitoylation and membrane attachment in Nmnat2 turnover. We conclude that Nmnat2 activity supports axon survival through a site of action distinct from Nmnat2 transport vesicles and that protein stability, a key determinant of axon protection, is enhanced by mutations that disrupt palmitoylation and dissociate Nmnat2 from these vesicles.

## Abstract 2

Modulation of the subcellular localization of the endogenous axon survival factor Nmnat2 boosts its axon protective capacity, suggesting a novel approach to delaying axon degeneration in neurodegenerative disease.

## Author Summary

Neurons are polarized cells that rely on bidirectional transport to deliver thousands of cargos between the cell body and the most distal ends of their axons. One cargo that is of particular importance is the NAD-synthesising enzyme Nmnat2. This surprisingly unstable protein is produced in the cell body and its constant supply into axons is required to keep them alive. If this supply is interrupted, Nmnat2 levels in the distal axon drop below a critical threshold, leading to axon degeneration. The rapid turnover of Nmnat2 contributes critically to the time course of axon degeneration. If its half-life could be extended, axons may be able to survive transient interruptions of its supply. In this study, we find that disruption of Nmnat2 localization to axonal transport vesicles increases both its half-life and its capacity to protect injured neurites. Specifically, association of Nmnat2 with transport vesicles reduces it stability by making it vulnerable to ubiquitination and proteasome-mediated degradation. These findings suggest that modulation of the subcellular localization of Nmnat2 on transport vesicles could serve as a potential avenue for therapeutic treatment of axon degeneration.

## Introduction

The chimeric fusion protein Wld^S^ (Entrez Gene ID 22406) affords robust protection of injured axons in vitro and in vivo [1],[2] and extends axon survival in several disease models [3]–[9]. The Wld^S^ protein incorporates full-length Nmnat1 (Entrez Gene ID 66454), an NAD synthesising enzyme whose enzymatic activity is necessary for the protective effect of Wld^S^ [10],[11]. Additionally Wld^S^ harbours an N-terminal region that causes its partial re-distribution from the nucleus to an axoplasmic localization that is necessary for axon protection in vivo [1],[10]–[12]. We recently identified the related NAD-synthetic enzyme Nmnat2 (Entrez Gene ID 226518) as a labile, endogenous axon survival factor whose constant supply from cell bodies into axons is required for axon survival in primary culture. Specific depletion of Nmnat2 causes neurite degeneration without injury. After injury, endogenous Nmnat2 is depleted rapidly in the distal stump of neurites, initiating the process of Wallerian degeneration. If the more long-lived Wld^S^ protein is present in the axon, Nmnat2 is still depleted at the same rate; however, the NAD-synthetic enzyme activity of the stable Wld^S^ protein substitutes for that of Nmnat2, resulting in a significant delay of Wallerian degeneration [13].

Supporting a role of Nmnat2 in axon survival, strong overexpression of Nmnat2 delays Wallerian degeneration in vitro, and this protective effect is dependent on its enzymatic activity [13],[14]. Furthermore, Nmnat2 overexpression delays injury-induced axon degeneration in zebrafish [15] and alleviates neurodegeneration in the P301L mouse model of tauopathy [16]. Spontaneous synapse and axon loss in *Drosophila* lacking dNmnat, which can be partially rescued by murine Nmnat2, also supports a key role for endogenous axonal Nmnat activity in axon survival [17].

Neurons are extremely polarized cells with processes extending up to centimetres or even meters beyond the cell body. Moreover, in some neurons the axon constitutes over 99% of total cytoplasmic volume [18]. Despite growing evidence for the local, axonal synthesis and regulation of some proteins [19]–[21], many others appear to be synthesized only in the cell body and rely on axonal transport to reach their site of action in the axon or synapse. This supply process is a huge logistical challenge, and not surprisingly, any impairment affects axonal function or survival. Indeed, recent work has illustrated that there is a significant, early impairment in axonal transport in many neurodegenerative conditions and, for at least some of these, impaired axonal transport appears to cause the degenerative process [22].

Given that Nmnat2 is essential for axon maintenance [13] and its mRNA has not been found in axons [23]–[26], the delivery of this short-lived protein into axons is likely to limit axon survival when axonal transport is impaired through injury, aging, or disease. Although the Wld^S^ protein can overcome the need for Nmnat2, its clinical application faces the problem that this gain-of-function chimeric fusion protein is expressed only in Wld^S^ mice and a few strains of transgenic organisms. Instead, understanding and manipulating the delivery, turnover, or intra-axonal targeting of the endogenous survival factor, Nmnat2, is a more promising route to influence axon survival. Thus, it is important to understand how healthy neurons ensure a steady supply of Nmnat2 and the mechanisms that regulate its delivery, turnover, and activity in axons.

Nmnat2 localizes to vesicular structures and undergoes fast axonal transport in the neurites of primary culture neurons [13],[27]. Previous work showed that association of Nmnat2 with Golgi membranes in HeLa cells requires palmitoylation of the two adjacent cysteine residues C164/165. In the absence of these residues, Nmnat2 adopts a more diffuse, cytosolic localization [27],[28]. This palmitoylation site is located within the isoform-specific targeting and interaction domain (ISTID) of Nmnat2. ISTID regions are found in all three mammalian Nmnat isoforms and, in contrast to the more conserved core catalytic domains that make up the remainder of the proteins, are highly divergent between isoforms and are thus thought to account for the differential subcellular localizations of the Nmnats [28].

Here we report that the small, centrally located exon 6 is both necessary and sufficient for palmitoylation, stable membrane association, and vesicle-mediated delivery of Nmnat2 into axons. By manipulating its localization, we then test the hypotheses that these transport vesicles are the sites of Nmnat2 axon-protective action and that vesicular Nmnat2 is somehow protected from rapid turnover, enabling it to reach the ends of long axons before being degraded. Surprisingly, we find that a diffuse, nonvesicular localization enhances Nmnat2 axon protection through increased protein stability. Our results support a model in which Nmnat2 subcellular localization regulates its turnover and protective capacity and suggest a site of action distinct from its transport vesicles.

## Results

### Nmnat2 Is Delivered to Axons With a Golgi-Derived Vesicle Population

**Figure 1:**
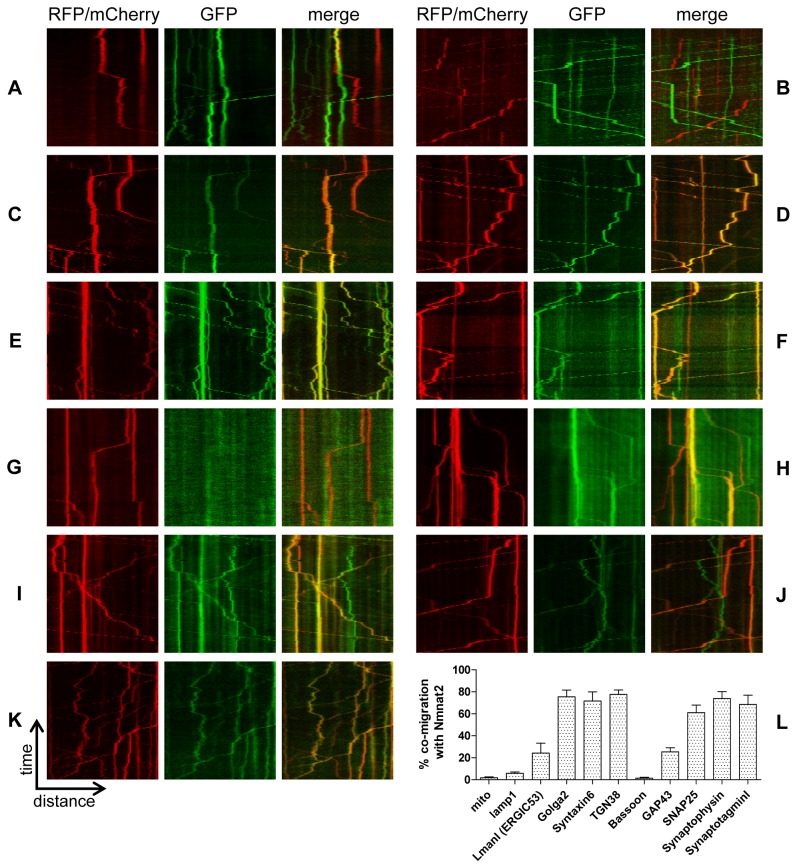
Nmnat2 co-migrates with markers for trans-Golgi and synaptic vesicles. Co-migration was analysed using kymographs (time-distance graphs) obtained from live-cell imaging of neurites from primary culture SCG neurons. Representative kymographs from neurites co-labelled with Nmnat2-EGFP and (A) mito-tagRFP, (B) lamp1-RFP, or Nmnat2-mCherry and (C) LmanI-EGFP, (D) Golga2-EGFP, (E) Syntaxin6-EGFP, (F) TGN38-EGFP, (G) GFP-Bassoon, (H) GAP43-EGFP, (I) SNAP25b-EGFP, (J) Synaptophysin-EGFP, and (K) SynaptotagminI-EGFP. (L) Quantification of co-migration. The quantification shown for each construct represents the percentage of moving Nmnat2-labelled vesicles that were also labelled by the relevant marker. Error bars indicate SEM.

Previously, we reported that, in primary culture neurons, Nmnat2-EGFP is transported in particulate structures with an anterograde bias and at velocities in the range of fast axonal transport [13]. To shed light on the identity of this trafficking organelle, we utilized dual-colour, live-cell imaging of primary culture superior cervical ganglia (SCG) neurons to visualize the co-transport of Nmnat2 with established axonal transport cargos and organelle markers. Co-migration of two fluorescent markers was quantified using kymographs (see Materials and Methods), counting only moving particles. We detected significant co-migration of Nmnat2 with several Golgi network markers (Golga2, Syntaxin6, TGN38; Figure 1D–F, L). Similarly, high levels of co-migration were observed for synaptic vesicle markers (SNAP25, Synaptophysin, SynaptotagminI; Figure 1I–K, L). Interestingly, however, no significant co-migration was found for mitochondria (Figure 1A, L), lysosomes (lamp1; Figure 1B, L), or active zone precursor vesicles (Bassoon; Figure 1G, L), and only partial co-migration was detected for a marker of the ER-Golgi intermediate compartment (ERGIC) (ERGIC53/LmanI; Figure 1C, L). Thus, Nmnat2 undergoes fast axonal transport in a Golgi-derived vesicle population that overlaps with synaptic vesicle precursors and is distinct from mitochondria, lysosomes, active zone precursor vesicles, and the ERGIC.

### Nmnat2 Exon 6 Encodes Sequences Sufficient for Stable Membrane Association and Axonal Transport

**Figure 2:**
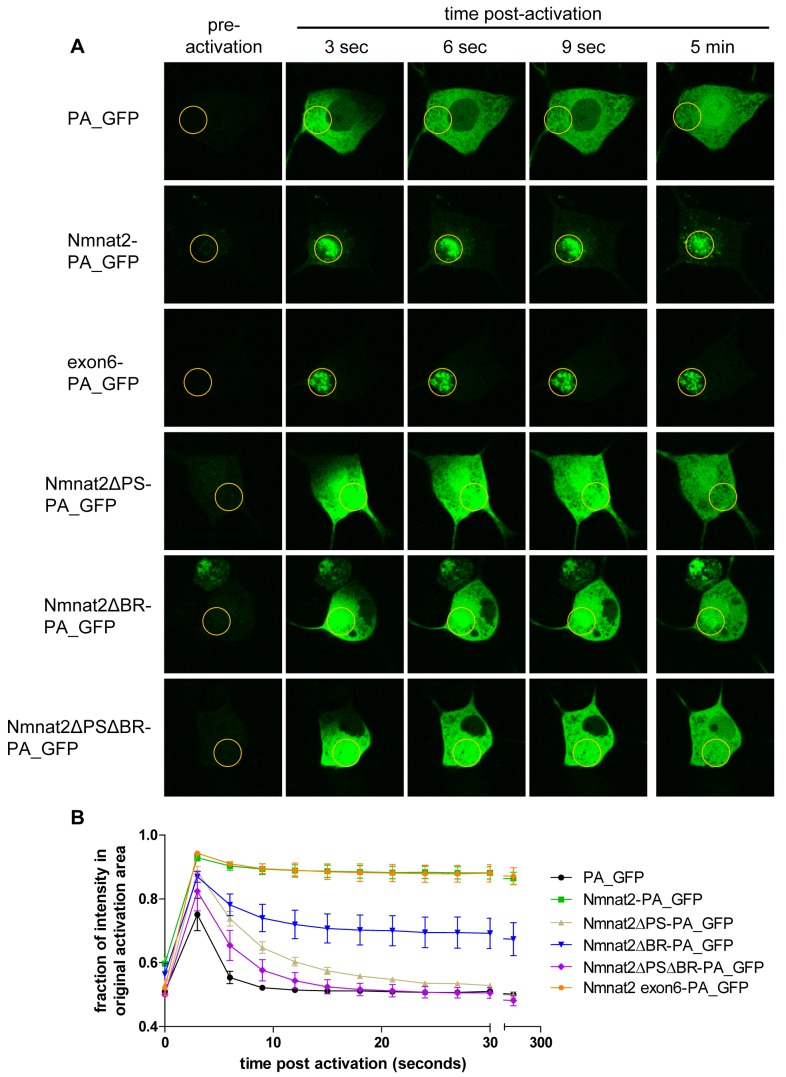
Exon 6 sequences are necessary and sufficient for Nmnat2 membrane association. (A) Use of photoactivatable GFP (PA_GFP) fusion proteins to study Nmnat2 membrane association. Time course of a representative cell body of an SCG primary culture neuron injected with each indicated construct is shown. The region of activation is marked by an orange circle in each image. (B) Quantification of protein mobility. Shown is the percentage of fluorescence that remains in the originally activated area compared to a non activated area of equal size elsewhere in the cell body. Error bars indicate SEM.

Next, we tested whether the reported mechanism of Golgi-targeting in HeLa cells [27],[28] applies to neuronal cell bodies and vesicle targeting in axons. In order to define the requirements for membrane association in neurons, we used a photoactivation assay in SCG cell bodies. Photoactivatable GFP (PA_GFP [29]) was fused to variant Nmnat2 sequences and microinjected along with an mCherry marker to identify injected cells. PA_GFP was activated in a small region of the cell body and the pool of activated PA_GFP followed over time. For quantification, we compared the fraction of fluorescence intensity that remained in the activated area with a non activated area elsewhere in the cell body (excluding the nucleus). Activated PA_GFP alone diffused very rapidly throughout the cell body, resulting in an even distribution of fluorescence after about 5–10 s (Figure 2). In contrast, Nmnat2-PA_GFP was retained within the originally activated area, suggesting strong membrane association that was stable over the course of the experiment (Figure 2; Table 1). This indicates that the majority of Nmnat2 is stably membrane-bound and most of the spread of fluorescence that did occur was slow and resulted from transport of vesicle-bound Nmnat2-PA_GFP out of the activated region (see Figure 2A).

**Table 1:** Best-fit values for exponential decay of PA_GFP diffusion in SCG cell bodies.

	y∞	k
PA_GFP	0.51	0.56 (0.42+)
Nmnat2-PA_GFP	0.88	0.23
Exon6-PA_GFP	0.88	0.23
Nmnat2ΔPS-PA_GFP	0.53	0.18
Nmnat2ΔBR-PA_GFP	0.69	0.22
Nmnat2ΔPSΔBR-PA_GFP	0.51	0.25

+ k-value adjusted for difference in molecular size between PA_GFP and Nmnat2-PA_GFP [30].

**Figure 3:**
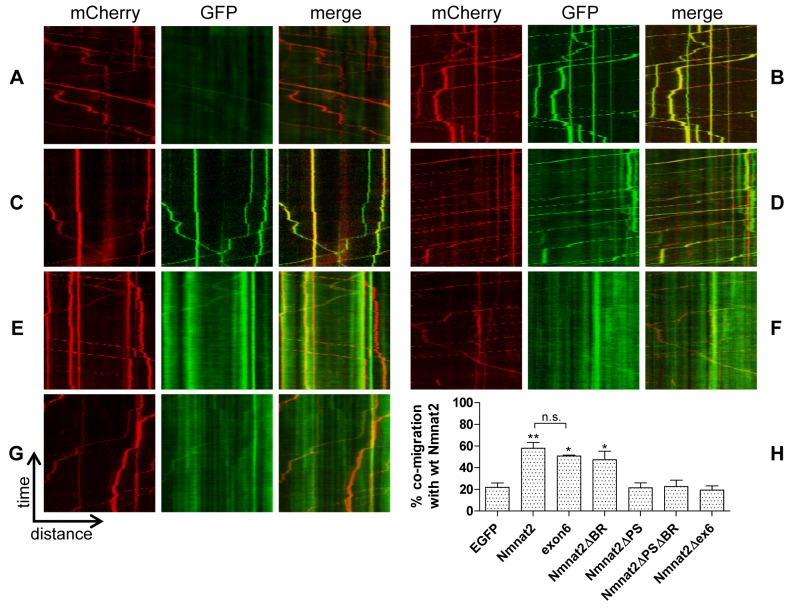
Cytosolic Nmnat2 mutants do not co-migrate with wild-type Nmnat2 in neurites. Representative kymographs from neurites co-labelled with Nmnat2-mCherry and (A) EGFP, (B) Nmnat2-EGFP, (C) exon6-EGFP, (D) Nmnat2ΔPS-EGFP, (E) Nmnat2ΔBR-EGFP, (F) Nmnat2ΔPSΔBR-EGFP, and (G) Nmnat2Δex6-EGFP. (H) Quantification of co-migration. The quantification shown for each construct represents the percentage of moving wild-type Nmnat2-labelled vesicles that were also labelled by the relevant marker. Error bars indicate SEM. * and ** indicate statistically significant difference compared to EGFP (* *p*<0.05, ** *p*<0.01, n.s. non-significant).

The C164/165 palmitoylation site is located at the centre of the 27 amino acids encoded by exon 6 of Nmnat2 (see Figure S1A for Nmnat2 primary structure with relevant regions highlighted). To test whether this exon is sufficient for membrane targeting, we created an exon6-PA_GFP construct and subjected it to the membrane association assay described above. Interestingly, we observed a very strong membrane association that was indistinguishable from that seen with full-length Nmnat2-PA_GFP (Figure 2; Table 1). This same sequence also targeted EGFP to transport vesicles in neurites that co-migrated with full-length Nmnat2-mCherry. The degree of co-migration was similar to that between Nmnat2-EGFP and Nmnat2-mCherry (Figure 3B, C, and H). Together, these results indicate that exon 6 encodes residues sufficient for efficient, stable membrane association and the resulting vesicular fast axonal transport of Nmnat2.

### The C164/165 Palmitoylation Site and Surrounding Basic Residues Are Required for Efficient Palmitoylation, Stable Membrane Association, and Axonal Transport of Nmnat2

We then confirmed the requirement for the C164/165 palmitoylation site for membrane association in neurons, using a C164S/C165S construct (Nmnat2ΔPS-PA_GFP; see Figure S1B for an overview of all mutant constructs used in this study). Photoactivation in SCG cell bodies resulted in a rapid spread of fluorescence, similar to PA_GFP alone (Figure 2), although quantification revealed that the spread was slightly slower than for PA_GFP, even when accounting for differences in molecular size (see k values in Table 1) [30], suggesting that other residues contribute weakly to membrane association.

GAP43 (Entrez Gene ID 14432), which associates with membranes through palmitoylation of a similar double-cysteine motif, also requires an adjacent group of basic residues for efficient and stable membrane association [31]–[33] and shows partial co-migration with Nmnat2 (Figure 1H, L). We tested whether a similar mechanism applies to Nmnat2 by mutating the five basic residues encoded by exon 6 (K151A, K155A, R162A, R167A, and R172A). This construct, Nmnat2ΔBR-PA_GFP, showed intermediate membrane association. A pool of diffusible material spread quickly throughout the cell (as seen with PA_GFP), but a significant portion of the signal remained in the originally activated area for the duration of the experiment, suggesting the presence of a pool of strongly membrane-bound material (as seen with Nmnat2-PA_GFP and exon6-PA_GFP) (Figure 2). These results suggest that, in addition to the palmitoylated cysteines themselves, basic residues that surround C164/165 are also required for efficient palmitoylation and membrane association. However, the mobility of the diffusible portion of Nmnat2ΔBR-PA_GFP was not significantly different from that of Nmnat2ΔPS-PA_GFP (Figure 2; Table 1). Accordingly, a double mutant, Nmnat2ΔPSΔBR-PA_GFP, also showed the same mobility as either of the single mutants, thus still exhibiting slower diffusion than PA_GFP alone.

To confirm that these changes in membrane association reflect the degree of palmitoylation of Nmnat2, we used radiolabelling to measure palmitate incorporation into wild-type and mutant Nmnat2 (Figure S2). In agreement with previous findings [27],[28] we found that FLAG-Nmnat2ΔPS loses all detectable palmitoylation. Interestingly, however, a small but significant portion of palmitate incorporation was maintained in FLAG-Nmnat2ΔBR as predicted by the membrane association assay, further supporting the idea that exon 6 basic residues are necessary to enable efficient palmitoylation and only a small amount of palmitoylation can occur in their absence. To further substantiate the role of palmitoylation in Nmnat2 membrane association, we treated SCG neurons with 2-Bromopalmitate (2-BP), a lipid-based inhibitor of palmitoylation. As predicted, treatment with 2-BP substantially reduced membrane association of wild-type Nmnat2-PA_GFP in the photoactivation assay (Figure S6A, B).

Next, we sought to test the effect of exon 6 mutations on axonal transport of Nmnat2. As expected, mutation of the palmitoylation site in Nmnat2ΔPS-EGFP led to a diffuse, nonvesicular distribution in neurites. We detected little co-migration with Nmnat2-mCherry (Figure 3E), which was not significantly different from EGFP alone (Figure 3A, H). Like other cytosolic proteins, nonspecific or transient membrane association may help deliver this protein to neurites in this system [34]. For Nmnat2ΔBR-EGFP, the amount of diffuse, nonvesicular fluorescence signal was also greatly increased, but we still observed significant co-migration with Nmnat2-mCherry (Figure 3D, H), consistent with the residual palmitoylated, membrane-bound component inferred from the photoactivation and palmitate labelling experiments. Nmnat2ΔPSΔBR-EGFP and Nmnat2Δex6-EGFP (lacking all of exon 6) were both similar to Nmnat2ΔPS-EGFP with no further reduction in co-migration with Nmnat2-mCherry (Figure 3F–H). Taken together, these results suggest that, within exon 6, both the palmitoylation site and surrounding basic residues are necessary for efficient, stable membrane association, and vesicular axonal transport of Nmnat2.

### Loss of Nmnat2 Vesicle Targeting Increases Its Axon Protective Capacity

**Figure 4:**
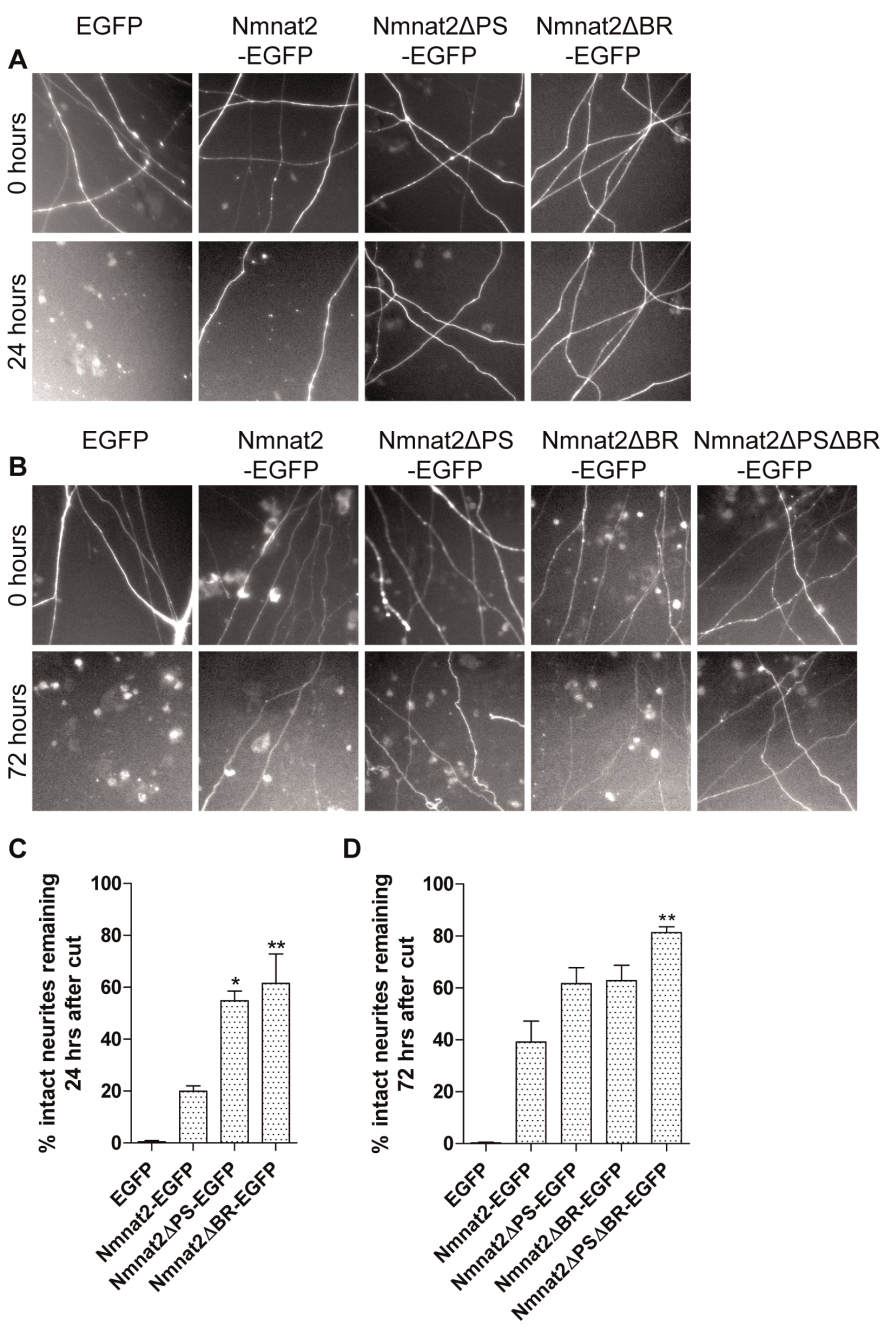
Cytosolic Nmnat2 mutants show moderate increase in neurite protective capacity. (A) Representative fields of view of distal primary culture SCG neurites 0 and 24 h after neurite cut, labelled by dsRed2 expression and injected with 0.001 µg/µl EGFP or the relevant Nmnat2-EGFP variant. (B) Representative fields of view of primary culture SCG neurites 0 and 72 h after neurite cut, labelled by dsRed2 expression and injected with 0.002 µg/µl EGFP or the relevant Nmnat2-EGFP variant. (C, D) Quantification of experiments in (A) and (B), respectively. Error bars indicate SEM. * and ** indicate statistically significant difference compared to wild-type Nmnat2 (* *p*<0.05, ** *p*<0.01).

We next tested the hypothesis that vesicle targeting is necessary for Nmnat2-mediated axon protection. We injected wild-type or variant Nmnat2-EGFP together with a DsRed2 fluorescent marker into SCG cell bodies and transected their neurites 48 h later. Due to its short half-life, Nmnat2-EGFP protects neurites for 24 h after transection only if strongly overexpressed. Surprisingly, however, both Nmnat2ΔPS-EGFP and Nmnat2ΔBR-EGFP preserved transected neurites significantly more strongly when only 0.001 µg/µl of DNA was injected (Figure 4A, C). Interestingly, the protective effects of these two mutations were additive. At 0.002 µg/µl, Nmnat2ΔPSΔBR-EGFP showed strongly preserved neurites up to 72 h, significantly more than either single mutant (Figure 4B, D). To rule out any influence of the EGFP tag on these results, we also found that untagged Nmnat2ΔPSΔBR protects neurites significantly better than untagged wild-type Nmnat2 (0.01 µg/µl; Figure S3). Thus, vesicle association is dispensable for Nmnat2-mediated neurite protection in primary culture, and missense mutations disrupting vesicle association boost Nmnat2 axon protective capacity to a modest but significant degree.

**Figure 5:**
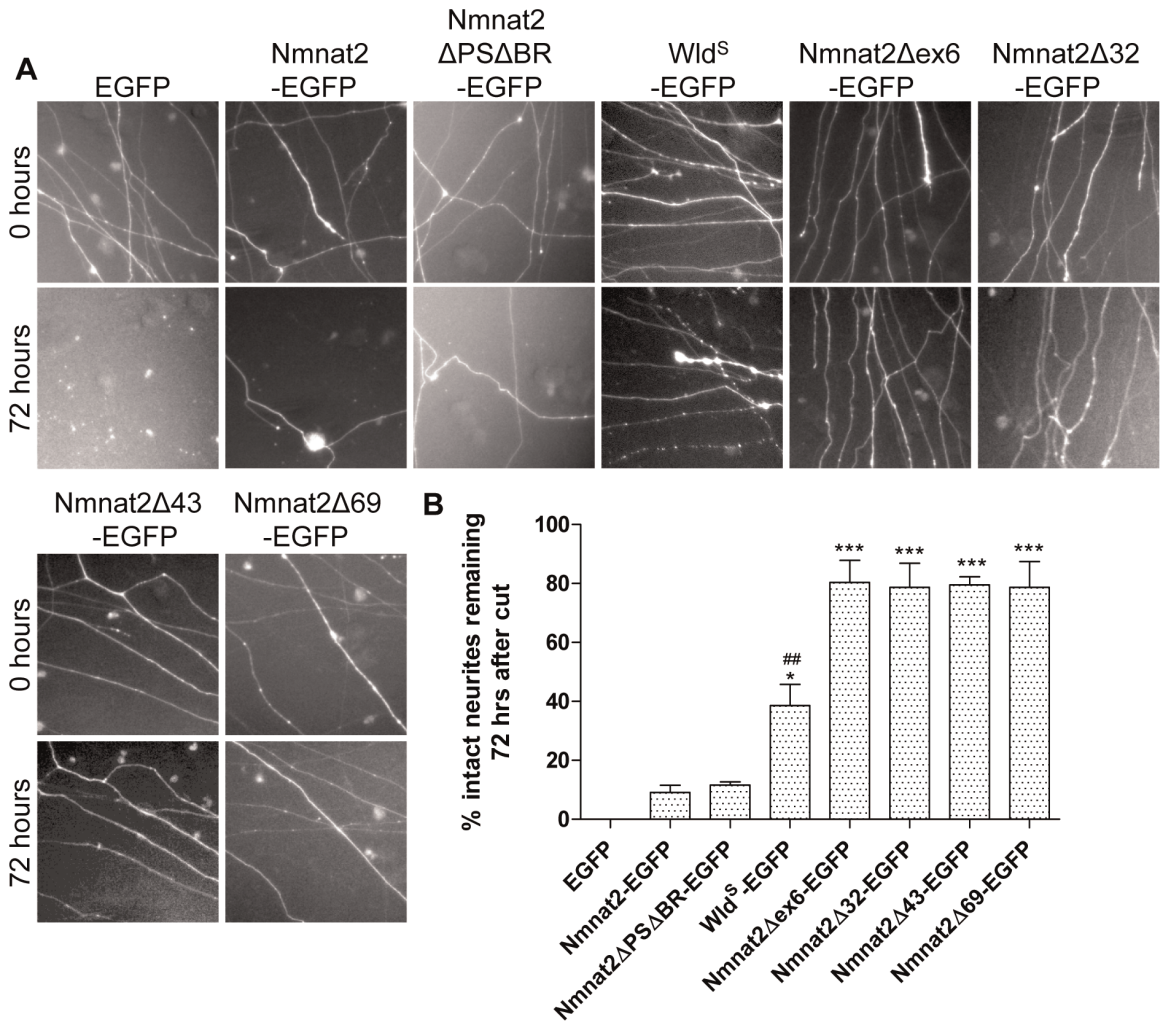
Nmnat2 deletion mutants show substantial neurite protection up to 72 h. (A) Representative fields of view of distal primary culture SCG neurites 0 and 72 h after neurite cut, labelled by dsRed2 expression and injected with 0.0005 µg/µl EGFP or the relevant Nmnat2-EGFP variant or Wld^S^-EGFP. (B) Quantification of experiment shown in (A). Error bars indicate SEM. * and *** indicate statistically significant difference compared to wild-type Nmnat2 (* *p*<0.05, *** *p*<0.001). ## indicates statistically significant difference compared to deletion mutants (## p<0.01).

These surprising findings prompted us to investigate the effects on axon protection of deletions within and around exon 6. In particular, three deletion mutants were recently reported to retain enzyme activity (Nmnat2Δ32-EGFP, Nmnat2Δ43-EGFP, Nmnat2Δ69-EGFP [35]). Remarkably, all these deletion mutants and a mutant lacking exon 6 only (Nmnat2Δex6-EGFP) protected neurites far more strongly than any of the missense constructs above. Even microinjection of very low DNA concentrations (0.0005 µg/µl) preserved around 80% of neurites for 72 h after transection (Figure 5), compared with less than 10% for Nmnat2-EGFP or Nmnat2ΔPSΔBR-EGFP at this concentration. Surprisingly, this even exceeds the level of protection achieved by Wld^S^-EGFP at this concentration (around 30% intact neurites at 72 h), illustrating the very strong enhancement of axon protective capacity in these mutants (Figure 5). The presence of the EGFP tag was not necessary for the observed increase in protection (Figure S4). To rule out the possibility that deletion of exon 6 induces a novel gain-of-function in Nmnat2 that is independent of its NAD-synthesis activity, we introduced an enzyme-dead mutation into Nmnat2Δex6. His24 is conserved in all three mammalian Nmnats and is critical for Nmnat2 NAD-synthesis activity as well as its ability to protect axons after cut [14],[36],[37]. Convincingly, a Nmnat2Δex6H24D enzyme-dead mutant did not protect neurites after injury (Figure S5), although we found that this mutation also reduces the stability of Nmnat2Δex6H24D (unpublished data). Together, these findings suggest that, in addition to the palmitoylation site and surrounding basic residues, other exon 6 residues influence the axon protective capacity of Nmnat2, while the additional sequences deleted outside of exon 6 in Nmnat2Δ32, Nmnat2Δ43, and Nmnat2Δ69 appear to have little further effect.

### Increased Neurite Protection Is Associated With Increased Protein Stability and Reduced Ubiquitination

**Figure 6:**
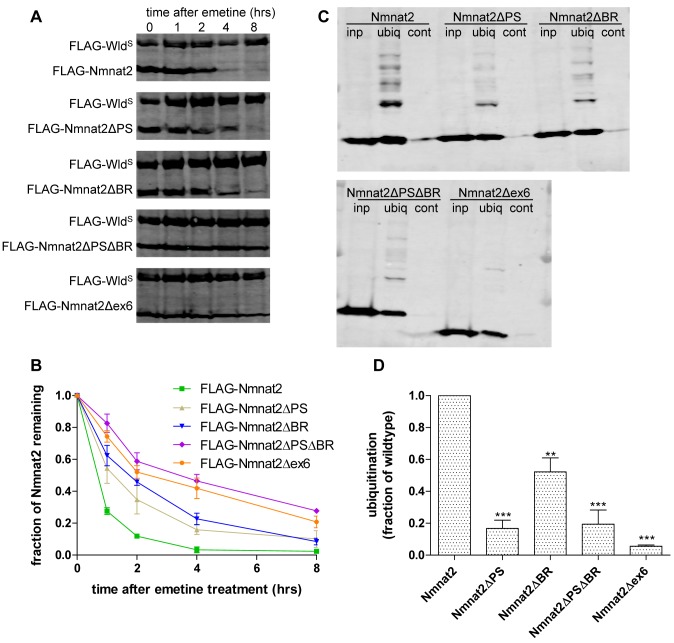
Cytosolic Nmnat2 mutants exhibit increased protein stability and reduced levels of ubiquitination. (A) Representative Western blots of HEK293 cells co-transfected with FLAG-Wld^S^ and the indicated FLAG-Nmnat2 variant. Twenty-four hours after transfection, cells were treated with 10 µM emetine for the amount of time indicated after which samples were processed for SDS-PAGE and Western blot using anti-FLAG antibody. (B) Quantification of Nmnat2 turnover after emetine treatment. For each sample and time point, the amount of FLAG-Nmnat2 remaining was normalised to FLAG-Wld^S^ as an internal control. Error bars indicate SEM. (C) Representative Western blots of GST-Dsk2 pulldown assay. HEK293 cells expressing a FLAG-Nmnat2 variant were lysed (inp. – total input) and ubiquitinated proteins were immunoprecipitated using GST-Dsk2 bound to glutathione beads (ubiq.). GST-fused mutant Dsk2 was used for control pulldown (cont.). Eluted proteins were processed for SDS-PAGE and analysed by Western blot using anti-FLAG antibody. (D) Quantification of ubiquitination assay. For each Nmnat2 variant, the total amount of ubiquitinated FLAG-Nmnat2 was normalised to total input. Error bars indicate SEM. ** and *** indicate statistically significant difference compared to wild-type Nmnat2 (** *p*<0.01*** *p*<0.001).

Next, we sought to identify the mechanism by which these mutations increase axon protective capacity. As the short half-life of Nmnat2 limits survival of injured axons [13], we decided to investigate protein stability using an emetine chase assay. HEK293 cells transiently expressing FLAG-Nmnat2 or one of its mutant forms were treated with 10 µM emetine to inhibit protein synthesis. Nmnat2 turnover was then measured as the rate of decline of the FLAG-Nmnat2 signal over time relative to a more stable control (FLAG-Wld^S^) [13]. We had envisaged that vesicular Nmnat2 may be relatively stable, allowing it to reach the ends of long axons, and in dynamic equilibrium with a less stable, but perhaps more active cytosolic form. However, all mutations disrupting membrane targeting were found to increase protein stability (Figure 6A, B; Table 2). Moreover, the FLAG-Nmnat2ΔPSΔBR double mutant showed significantly higher protein stability than either FLAG-Nmnat2ΔPS or FLAG-Nmnat2ΔBR, supporting a model in which an increase in protein stability contributes to the increase in protective capacity observed for these missense mutations. Intriguingly, however, the very strongly protective Nmnat2Δex6 construct was no more stable than Nmnat2ΔPSΔBR (Figure 6A, B; Table 2), suggesting that factors other than protein stability underlie the further increase in its protective capacity.

**Table 2:** Best-fit values for Nmnat2 wild-type and mutant half-lives.

	t½ (h)
FLAG-Nmnat2	0.6
FLAG-Nmnat2 + 2-BP	1.1
FLAG-Nmnat2ΔPS	1.3
FLAG-Nmnat2ΔBR	1.8
FLAG-Nmnat2ΔPSΔBR	3.6
FLAG-Nmnat2ΔPSΔBR-NterTGN	1.0
FLAG-Nmnat2ΔPSΔBR-NterMOM	0.9
FLAG-Nmnat2Δex6	3.4
FLAG-Nmnat2Δex6-NterTGN	0.9

As Nmnat2 degradation is blocked by proteasome inhibitor MG132 [13], we then asked whether Nmnat2 becomes ubiquitinated and whether these stabilizing mutations reduce ubiquitination. Wild-type and mutant FLAG-Nmnat2 were overexpressed in HEK293 cells, and the K48-specific ubiquitin binding domain of Dsk2 was bound to Glutathione-Sepharose beads and used to immunoprecipitate ubiquitinated proteins. An inactive mutant form of the Dsk2 UBA was used as a control [38],[39], and 20 µM MG132 was added 6 h prior to cell lysis to increase the abundance of ubiquitinated proteins. FLAG-Nmnat2 produced a clear ladder of ubiquitinated products (Figure 6C), and all missense mutants and Nmnat2Δex6 showed significantly less ubiquitination (Figure 6C, D). Thus, reduced ubiquitination is likely to contribute to the increased stability and protective capacity of these mutants.

### Vesicle Localization Is Sufficient to Promote Nmnat2 Ubiquitination and Lower Axon Protective Capacity

**Figure 7:**
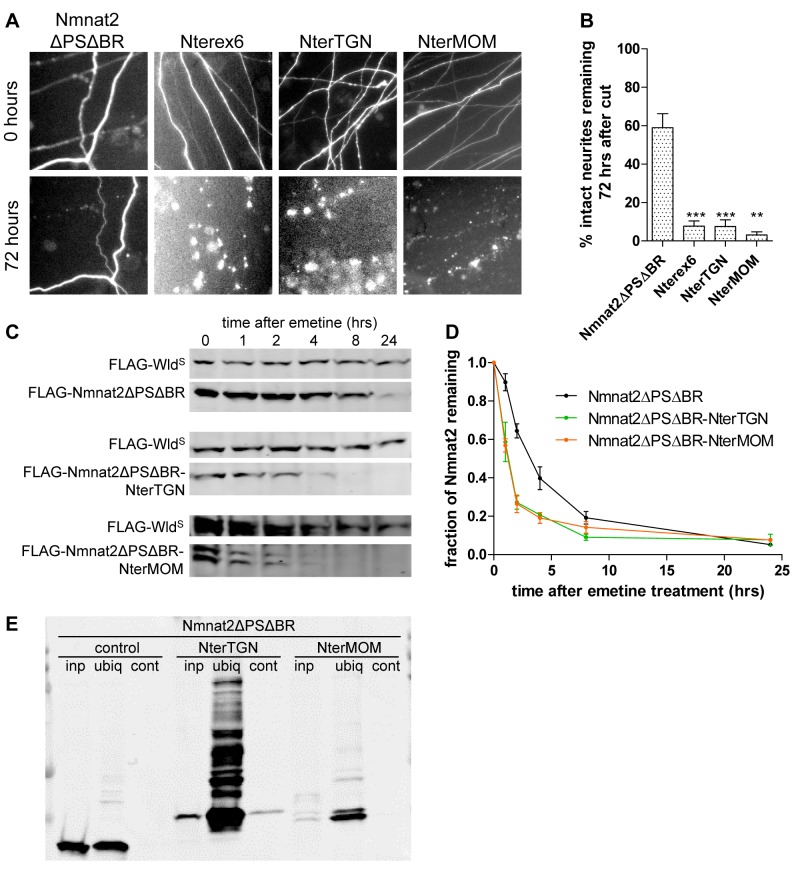
Re-targeting to membranes by N-terminal tags reduces neurite protection by Nmnat2ΔPSΔBR through effects on ubiquitination and protein stability. (A) Representative fields of view of distal primary culture SCG neurites 0 and 72 h after neurite cut, labelled by dsRed2 fluorescence and injected with 0.001 µg/µl of Nmnat2ΔPSΔBR-PA_GFP or one of its N-terminally membrane targeted variants (Nmnat2ΔPSΔBR-Nterex6-PA_GFP (Nterex6), Nmnat2ΔPSΔBR-NterTGN-PA_GFP (NterTGN), and Nmnat2ΔPSΔBR-NterMOM-PA_GFP (NterMOM). (B) Quantification of experiment shown in (A). Error bars indicate SEM. ** and *** indicate statistically significant difference compared to Nmnat2ΔPSΔBR (** *p*<0.01, *** *p*<0.001). (C) Representative Western blots of HEK293 cells co-transfected with FLAG-Wld^S^ and FLAG-Nmnat2ΔPSΔBR or one of its N-terminally re-targeted variants (FLAG-Nmnat2ΔPSΔBR-Nterex6 (Nterex6), FLAG-Nmnat2ΔPSΔBR-NterTGN (NterTGN), and FLAG-Nmnat2ΔPSΔBR-NterMOM (NterMOM)). Twenty-four hours after transfection, cells were treated with 10 µM emetine for the amount of time indicated after which samples were processed for SDS-PAGE and Western blot using anti-FLAG antibody. (D) Quantification of Nmnat2ΔPSΔBR turnover after emetine treatment. For each sample and time point the amount of FLAG-Nmnat2ΔPSΔBR remaining was normalised to FLAG-Wld^S^ as an internal control. Error bars indicate SEM. (E) Representative Western blot of GST-Dsk2 pulldown assay. HEK293 cells expressing a FLAG-Nmnat2ΔPSΔBR variant were lysed (inp. – total input), and ubiquitinated proteins were immunoprecipitated using GST-Dsk2 bound to glutathione beads (ubiq.). GST-fused mutant Dsk2 was used for control pulldown (cont.). Eluted proteins were processed for SDS-PAGE and analysed by Western blot using anti-FLAG antibody.

Based on these results, we hypothesized that palmitoylation and vesicle association cause wild-type Nmnat2 to become destabilised through increased levels of ubiquitination. In contrast, the nonvesicular, cytosolic location of the Nmnat2 mutants reduces ubiquitination and increases protein stability and protective capacity. The Nmnat2ΔPS data, and strongly enhanced protective capacity of Nmnat2Δex6 over Nmnat2ΔPSΔBR, indicate that the observed effects do not just reflect removal of lysine residues. In agreement with this model, inhibiting palmitoylation directly with 2-BP resulted in reduced levels of ubiquitination on FLAG-Nmnat2 (Figure S6C, D). Furthermore, 2-BP treatment enhanced the half-life of FLAG-Nmnat2 in the emetine chase assay (Figure S6E, F). Based on this, we predicted that a cytosolic, stable Nmnat2 mutant with increased protective capacity (such as Nmnat2ΔPSΔBR) would revert to a wild-type behaviour when re-targeted to vesicles. To test this, we first attached sequences to the N-terminus of Nmnat2ΔPSΔBR-PA_GFP that would re-target it to the same Golgi-derived vesicle population as wild-type Nmnat2. For this, we used exon 6 of Nmnat2 (Nmnat2ΔPSΔBR-Nterex6-PA_GFP) or the signal peptide and transmembrane domain of TGN38 (Nmnat2ΔPSΔBR-NterTGN-PA_GFP). These constructs were stably targeted to membranes as assessed by the photoactivation assay (Figure S7A, B), and as predicted, re-targeting significantly reduced the ability to protect injured neurites (Figure 7A, B). We also confirmed that Nmnat2ΔPSΔBR-NterTGN showed substantial levels of ubiquitination that were not detectable in Nmnat2ΔPSΔBR (Figure 7E) and that the protein stability of Nmnat2ΔPSΔBR-NterTGN was significantly reduced as expected (Figure 7C, D).

**Figure 8:**
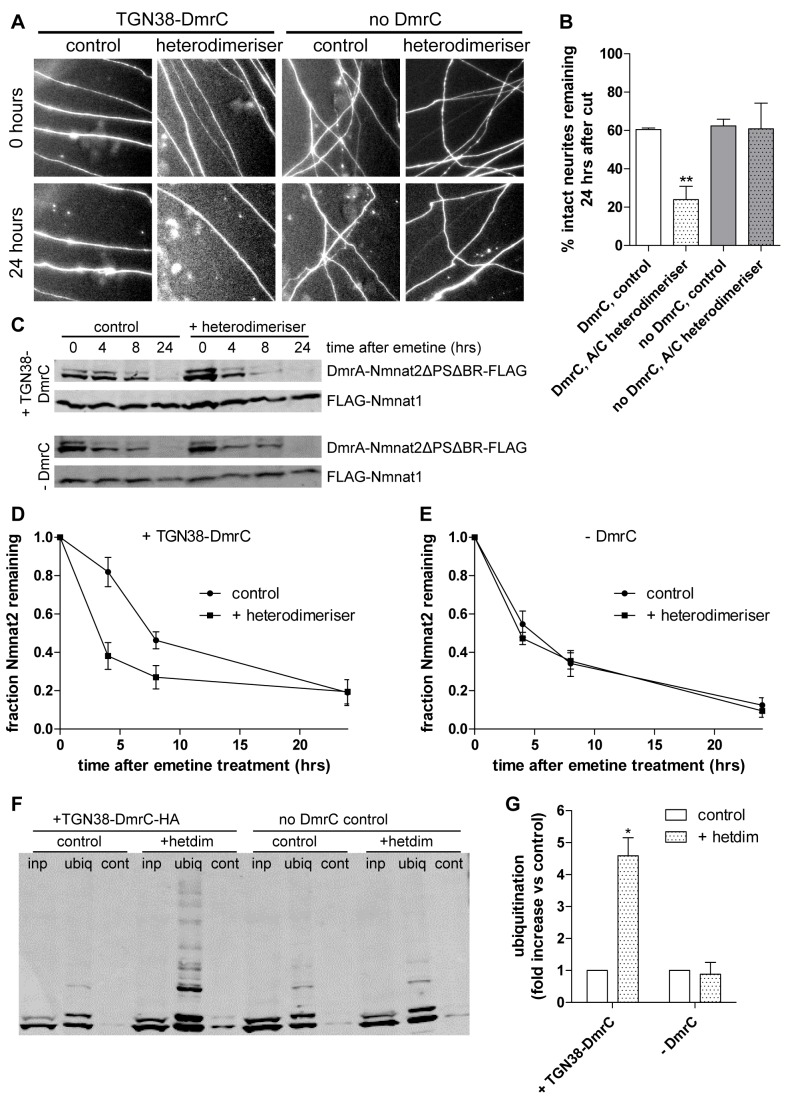
Membrane re-targeting through heterodimerisation causes ubiquitination, reduced protein stability, and impaired neurite protection by Nmnat2ΔPSΔBR. (A) Representative fields of view of distal primary culture SCG neurites 0 and 24 h after neurite cut, labelled by dsRed2 fluorescence and injected with 0.002 µg/µl of DmrA-Nmnat2ΔPSΔBR-PA_GFP plus 0.01 µg/µl TGN38-DmrC-HA or 0.001 µg/µl of DmrA-Nmnat2ΔPSΔBR-PA_GFP without any TGN38-DmrC-HA present. Eight hours before neurite cut, 500 nM of A/C heterodimeriser was added to relevant cultures. (B) Quantification of experiment shown in (A). Error bars indicate SEM. ** indicates statistically significant difference compared to control (** *p*<0.01). (C) Representative Western blots of HEK293 cells co-transfected with DmrA-Nmnat2ΔPSΔBR-FLAG, FLAG-Nmnat1, and TGN38-DmrC-HA (+TGN38-DmrC) or empty pCMV-Tag4A vector (-DmrC). To induce heterodimerisation, 500 µM A/C heterodimeriser were added to the appropriate wells. Twenty-four hours after transfection, cells were treated with 10 µM emetine for the amount of time indicated, after which samples were processed for SDS-PAGE and Western blot using anti-FLAG antibody. (D) Quantification of DmrA-Nmnat2ΔPSΔBR-FLAG turnover after emetine treatment when co-transfected with TGN38-DmrC-HA. For each sample and time point, the amount of DmrA-Nmnat2ΔPSΔBR-FLAG remaining was normalised to FLAG-Nmnat1 as an internal control. Error bars indicate SEM. (E) Quantification of DmrA-Nmnat2ΔPSΔBR-FLAG turnover after emetine treatment in the absence of TGN38-DmrC-HA. For each sample and time point, the amount of DmrA-Nmnat2ΔPSΔBR-FLAG remaining was normalised to FLAG-Nmnat1 as an internal control. Error bars indicate SEM. (F) Representative Western blot of GST-Dsk2 pulldown assay. HEK293 cells expressing DmrA-Nmnat2ΔPSΔBR-FLAG and TGN38-DmrC-HA and maintained in the absence (control) or presence (+heterodimeriser) of 500 µM A/C heterodimeriser were lysed (inp – total input), and ubiquitinated proteins were immunoprecipitated using GST-Dsk2 bound to glutathione beads (ubiq). GST-fused mutant Dsk2 was used for control pulldown (cont). Eluted proteins were processed for SDS-PAGE and analysed by Western blot using anti-FLAG antibody. (G) Quantification of ubiquitination assay. For each condition, the total amount of ubiquitinated DmrA-Nmnat2ΔPSΔBR-FLAG was normalised to total input. Error bars indicate SEM. *** indicates statistically significant difference compared to control (*** *p*<0.001).

While these results suggest that re-targeting cytosolic Nmnat2 to membranes reverts its stability and protective capacity to lower levels as expected, we cannot rule out the possibility that the N-terminal sequences have a direct effect on Nmnat2 stability. To address this issue, we used a commercially available heterodimerisation system (iDimerize, Clontech), in which two proteins tagged with DmrC and DmrA domains, respectively, undergo heterodimerisation after addition of a soluble “A/C heterodimeriser” compound [40]. We used TGN38-DmrC-HA to provide the membrane anchor for re-targeting of cytosolic DmrA-Nmnat2ΔPSΔBR-PA_GFP based on the strong co-migration of TGN38 with Nmnat2 (see Figure 1). Thus, this system overcomes the abovementioned limitation of the N-terminal targeting sequence as all that is required to induce membrane re-targeting is addition of the small molecule heterodimeriser compound. The photoactivation assay confirmed successful re-targeting, as mobility of DmrA-Nmnat2ΔPSΔBR-PA_GFP was significantly reduced after addition of heterodimeriser, albeit less strongly than using the N-terminal targeting sequence above (Figure S8). Correspondingly, addition of heterodimeriser also significantly reduced the axon protective capacity of DmrA-Nmnat2ΔPSΔBR-PA_GFP, but only in the presence of TGN38-DmrC-HA (Figure 8A, B). Even though the reduction in protective capacity observed in response to N-terminal re-targeting was stronger than that achieved by heterodimerisation, this was reflected in a lower degree of membrane re-targeting in the heterodimerisation system (compare Figure S7A, B and Figure S8). Additionally, we confirmed that re-targeting of cytosolic Nmnat2ΔPSΔBR to membranes through heterodimerisation resulted in increased levels of ubiquitination (Figure 8F, G) and a decreased protein half-life (Figure 8C–E), confirming the results obtained with N-terminally re-targeted mutants above.

At this point, it is interesting to ask whether the observed changes after Nmnat2 re-targeting arise from a special property of the vesicle membranes that Nmnat2 exon 6 and TGN38 target to, or whether they reflect a more general effect of Nmnat2 membrane association. To test this, we attached the mitochondrial outer membrane anchor (a.a. 1–37) of TOM20 to the N-terminus of Nmnat2ΔPSΔBR (Nmnat2ΔPSΔBR-NterMOM). Note that, as with Nmnat2ΔPSΔBR-NterTGN, the Nmnat2 portion of this construct faces the cytosol. The NterMOM tag led to stable membrane association in the photoactivation assay (Figure S7A, B). To confirm targeting to mitochondria, we co-stained neurons expressing Nmnat2ΔPSΔBR-NterMOM-PA_GFP with MitoTracker dye and observed largely overlapping staining patterns (Figure S7C). We then subjected Nmnat2ΔPSΔBR-NterMOM to the ubiquitination assay and found no evidence for increased levels of ubiquitination relative to Nmnat2ΔPSΔBR (Figure 7E). This suggests that the induction of ubiquitination after membrane attachment is indeed specific for transport vesicle membranes. Despite the absence of detectable ubiquitination, however, we found Nmnat2ΔPSΔBR-NterMOM to be destabilized relative to Nmnat2ΔPSΔBR (Figure 7C, D), which resulted in a loss of protective capacity (Figure 7A, B). These findings suggest that targeting to the mitochondrial outer membrane destabilizes Nmnat2 through a mechanism distinct from that operating on transport vesicle membranes.

**Figure 9:**
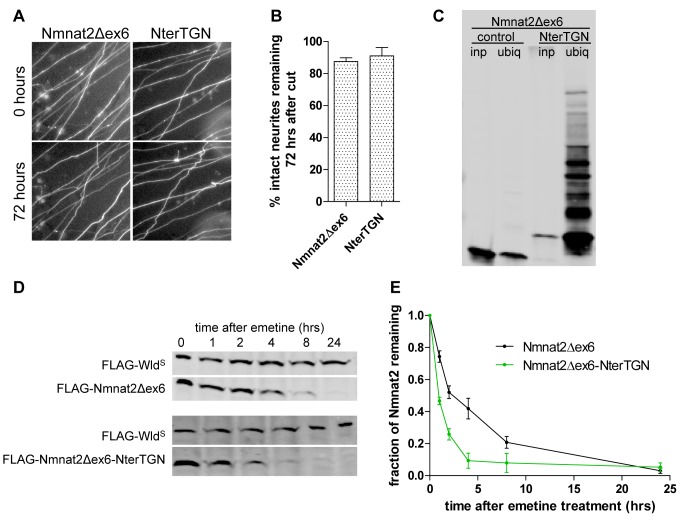
Membrane re-targeting through N-terminal TGN38 tag causes ubiquitination and destabilization but does not impair protective capacity of Nmnat2Δex6. (A) Representative fields of view of distal primary culture SCG neurites 0 and 72 h after neurite cut, labelled by dsRed2 expression and injected with 0.0005 µg/µl Nmnat2Δex6-EGFP or Nmnat2Δex6-NterTGN-EGFP. (B) Quantification of experiment shown in (A). Error bars indicate SEM. (C) Representative Western blot of GST-Dsk2 pulldown assay. HEK293 cells expressing FLAG-Nmnat2Δex6 or FLAG-Nmnat2Δex6-NterTGN were lysed (inp. – total input), and ubiquitinated proteins were immunoprecipitated using GST-Dsk2 bound to glutathione beads (ubiq.). GST-fused mutant Dsk2 was used for control pulldown (cont.). Eluted proteins were processed for SDS-PAGE and analysed by Western blot using anti-FLAG antibody. (D) Representative Western blot of HEK293 cells co-transfected with FLAG-Wld^S^ and FLAG-Nmnat2Δex6 or FLAG-Nmnat2Δex6-NterTGN. Twenty-four hours after transfection, cells were treated with 10 µM emetine for the amount of time indicated after which samples were processed for SDS-PAGE and Western blot using anti-FLAG antibody. (E) Quantification of Nmnat2Δex6 turnover after emetine treatment. For each sample and time point, the amount of FLAG-Nmnat2Δex6 remaining was normalised to FLAG-Wld^S^ as an internal control. Error bars indicate SEM.

As described above, deletion of exon 6 led to a very strong increase in Nmnat2 protective capacity without any further changes in stability with respect to Nmnat2ΔPSΔBR. To further explore this dissociation between protective capacity and protein stability, we re-targeted Nmnat2Δex6 to vesicle membranes using the N-terminal TGN38 tag. Nmnat2Δex6-NterTGN was stably targeted to membranes in the photoactivation assay (Figure S9) and showed increased ubiquitination (Figure 9C) and reduced protein stability (Figure 9D, E) with respect to Nmnat2Δex6. Interestingly, however, this did not affect its protective capacity, which remained indistinguishable from Nmnat2Δex6 up to 72 h after cut (Figure 9A, B). This finding suggests that the increase in protective capacity resulting from loss of exon 6 is sufficient to strongly delay degeneration even when only a low, residual level of protein remains.

## Discussion

Nmnat2 is required for axon survival and is the only confirmed endogenous Nmnat isoform in axons. However, its ability to promote axon survival is limited by its short half-life. We have identified a series of mutations that extend Nmnat2 half-life without disrupting enzyme activity and which significantly increase axon protection. For deletion mutants lacking exon 6 the efficacy even surpasses that of Wld^S^. Surprisingly, these changes arise when Nmnat2 targeting to a population of post-Golgi axonal transport vesicles is disrupted and are reversed when vesicle targeting is restored, indicating a nonvesicular site for the axon survival function of Nmnat2. The more stable and protective variants are less prone to ubiquitination through a mechanism likely to involve subcellular targeting and not just lysine availability. We identify cysteine-linked palmitoylation as the vesicle targeting mechanism and propose modulation of this targeting as a promising, novel therapeutic strategy for axonopathies.

We show that Nmnat2 axonal transport vesicles carry Golgi markers as well as synaptic vesicle markers. In contrast, we find no evidence of Nmnat2 undergoing co-transport with mitochondria. This suggests that Nmnat2 is involved in the regulation of cytosolic and not mitochondrial NAD metabolism in the axon, especially since mitochondria are not thought to take up cytosolic NAD under normal conditions [41]. Thus, any potential influence of Nmnat2 on mitochondrial function is likely to be mediated through indirect mechanisms. Furthermore, given that removal of Nmnat2 from its vesicles does not impair its protective capacity, it seems unlikely that Nmnat2 exists in close proximity to important downstream targets on its own transport vesicles. If this were the case, removal of Nmnat2 from this microenvironment would be expected to result in reduced levels of neurite protection. Instead, it appears that the regulation of overall cytosolic NAD metabolism by Nmnat2 is critical for axon survival.

Given the importance of axonal transport, surprisingly little is known about sequences targeting proteins to axons. The mechanism of Nmnat2 membrane association appears very similar to that reported for another axonally transported protein, GAP43. Both proteins lack a transmembrane domain or alternative membrane targeting structures and depend fully on palmitoylation of a double-cysteine motif for membrane association [27],[28],[42],[43]. For GAP43, it was reported that, in addition to the palmitoylated cysteine residues themselves, several adjacent basic amino acid residues are also necessary for efficient membrane association [31]–[33]. We found a similar mechanism to operate for Nmnat2. Our results suggest that basic amino acid residues surrounding the palmitoylation site in exon 6 are involved in mediating initial membrane contact and allow palmitoylation to establish stable membrane anchoring. In their absence, the level of palmitoylation is strongly reduced and membrane targeting of Nmnat2 is less efficient, resulting in a higher level of diffuse, soluble protein. However, once palmitoylation has occurred, membrane association seems to be as stable as for wild-type Nmnat2 over the time-scale of our analysis. This view is also supported by our finding that Nmnat2ΔBR, despite its increased level of diffuse fluorescence in neurites, is still associated with the correct population of transport vesicles. These similarities between GAP43 and Nmnat2 suggest a common underlying axonal targeting motif based on a dual-cysteine palmitoylation site and adjacent or surrounding lysine and arginine residues.

Palmitoylation regulates the axonal transport and subcellular sorting of several axonally delivered proteins [44] and is unique among the fatty-acid modifications in that it is readily reversible. It is now well established that palmitate cycling, the recurrent addition and removal of palmitate groups to a target protein, is an important regulatory mechanism in many cases [45]. Thus it is possible that endogenous Nmnat2 undergoes similar cycles of palmitoylation and depalmitoylation. If this is the case, a model could be envisaged in which Nmnat2 is palmitoylated and vesicle bound for the purpose of delivery over long distances into axons. Once in the axon, depalmitoylation could cause Nmnat2 to detach from vesicles and instead assume a diffuse, cytosolic localization in order to carry out its function in regulating axoplasmic NAD metabolism. Thus, palmitate cycling would effectively regulate the ratio of vesicle-bound to diffuse Nmnat2, and hence ultimately the axon protective capacity of Nmnat2. Modulation of the Nmnat2 palmitoylation-depalmitoylation cycle by targeting the relevant palmitoyltransferase and thioesterase enzyme(s) might hence present a useful tool to alter the course of axon degeneration.

We also found support for the hypothesis that increased protein stability underlies the mechanism by which diffuse, cytosolic Nmnat2 becomes more highly protective. Wild-type Nmnat2, which is mainly vesicle-bound, is very short-lived both in cell lines and in the neurites of primary culture neurons [13]. Here we found it to have a protein half-life of around 40 min, whereas soluble, cytosolic Nmnat2 mutants have a longer half-life. This means that while wild-type Nmnat2 is very rapidly depleted when its supply stops after neurite transection, cytosolic mutants with increased protein stability have an increased potency to protect neurites against degeneration, due to a combination of higher steady-state levels in the neurites before transection and a longer protein half-life after transection. Moreover, the reduced level of ubiquitination in these cytosolic mutants suggests that increased protein stability is a direct result of reduced turnover of Nmnat2 by the ubiquitin-proteasome system. This fits with our previous findings that inhibition of the ubiquitin proteasome system, which was shown to delay Wallerian degeneration [46], stabilizes endogenous Nmnat2 [13]. Thus our data support a model in which vesicle-bound Nmnat2 is unstable due to its high levels of ubiquitination, which in turn results in its rapid turnover and short protein half-life. Releasing Nmnat2 from its vesicles reduces ubiquitination and leads to a more stable protein with a higher axon protective capacity. Our results indicate that Nmnat2 can undergo ubiquitination on lysine residues outside of exon 6 as Nmnat2 mutants lacking exon 6 lysines (Nmnat2ΔPSΔBR and Nmnat2Δex6) can still be ubiquitinated when re-targeted to membranes.

Interestingly, this destabilising effect of palmitoylation-mediated membrane attachment contrasts with findings for several palmitoylated transmembrane domain proteins, including cell-surface receptors [47]–[50] and SNARE proteins [51], for which it was found that palmitoylation increases protein half-life through reduction of ubiquitin-proteasome mediated degradation. Our results indicate that the effect of palmitoylation on ubiquitination and protein stability might differ for proteins lacking transmembrane regions (such as Nmnat2). The mechanism by which palmitoylation-mediated vesicle-association causes high levels of ubiquitination in Nmnat2 is as yet unclear and will be the object of future studies. One interesting possibility is the localization of a relevant ubiquitin ligase to the surface of Nmnat2 transport vesicles. Such a vesicle-specific mechanism is supported by our finding that re-targeting cytosolic Nmnat2 mutants to mitochondrial outer membranes does not induce detectable ubiquitination. Alternatively, vesicular axonal transport may deliver Nmnat2 to parts of the axon with higher levels or activity of relevant elements of the ubiquitin proteasome system. A recently published study reported one such element regulating the turnover of *Drosophila* Nmnat (dNmnat) in axons. The *Drosophila* E3 ubiquitin ligase Highwire was found to be necessary and sufficient for rapid turnover of dNmnat, and of ectopically expressed mammalian Nmnat2, in the distal stump of injured axons. In its absence, dNmnat persists and degeneration is delayed [52]. Together with our findings, this suggests that ubiquitination regulates the course of axon degeneration both in mammals and *Drosophila* and that Nmnat2 axonal transport vesicles play an important role in bringing together dNmnat or Nmnat2 with their respective ubiquitin ligases.

Furthermore, our results indicate that subcellular localization and protein stability are not the only determinants of Nmnat2 axon protective capacity. Deletion of exon 6 dramatically increases Nmnat2-mediated neurite protection without any further increase in protein stability. Our findings with the enzyme-dead exon 6 deletion mutant suggest that this strong increase in protective capacity depends on Nmnat2 enzymatic activity. However, the reduced stability of this mutant means we cannot completely rule out the possibility that other, nonenzymatic mechanisms contribute to the rise in protective capacity. Interestingly, deletion of exon 6 overcomes the reduction in Nmnat2 protective capacity upon membrane re-targeting that was observed for point mutants. This rescue occurred despite the destabilising effects of membrane attachment, which were unchanged by the removal of exon 6. This suggests that exon 6 regulates Nmnat2 axon protective function through various mechanisms, which could include protein-protein interactions or additional posttranslational modifications.

In summary, we show that Nmnat2, normally the least axon protective of the three endogenous Nmnat isoforms due to its short half-life, can be converted to a highly protective molecule by disrupting its targeting to axonal transport vesicles. While the importance of these vesicles for long-range axonal trafficking is clear, we suggest that Nmnat2 must dissociate to carry out its axon survival function optimally. We also propose that cytosolic NAD metabolism is central to the axon survival mechanism. Our data establish the principle that Nmnat2 can be modified to promote axon survival and highlight modulation of its palmitoylation state as a route to achieve this. Unlike Wld^S^ or other Nmnats, this approach utilizes a protein already identified in wild-type axons, raising the attractive prospect of converting an endogenous axonal protein into one with a protective capacity that matches or even exceeds that of Wld^S^.

## Materials and Methods

### DNA Constructs

Nmnat2-EGFP, FLAG-Nmnat2, and FLAG-Wld^S^ constructs were described previously [13]. Wld^S^-EGFP was created by insertion of the Wld^S^ coding sequence into the MCS of pEGFP-NI vector (Clontech). Nmnat2-mCherry was created by replacing the EGFP coding sequence of Nmnat2-EGFP with the mCherry coding sequence from pmCherry-NI (Clontech). Nmnat2ΔPS-EGFP, Nmnat2ΔBR-EGFP, and Nmnat2ΔPSΔBR-EGFP were created from Nmnat2-EGFP using the QuikChange II Site Directed Mutagenesis Kit (Stratagene) according to the manufacturer's instructions. Nmnat2Δex6-EGFP was created by PCR amplification of the Nmnat2-EGFP vector excluding exon 6 and introduction of a SacII restriction site to allow vector re-ligation. FLAG-tagged, untagged, and PA_GFP tagged Nmnat2 wild-type or mutants were created by insertion of the appropriate Nmnat2 mutant into the MCS of pCMV-Tag2A (Stratagene), pCMV-Tag4A (Stratagene), and pPAGFP-NI [29] (Addgene plasmid 11909) vectors, respectively. Exon6-EGFP and Exon6-PA_GFP constructs were created by PCR of exon6 from Nmnat2 and insertion into the MCS of pEGFP-NI and pPAGFP-NI vectors. DmrA-Nmnat2ΔPSΔBR-PA_GFP was created by PCR amplification of the DmrA coding sequence from pHet-NucI vector (Clontech) and insertion at the N-terminus of Nmnat2ΔPSΔBR-PA_GFP. For the TGN38-DmrC-HA construct, the DmrC-HA coding sequence was PCR amplified from the pHET-1 vector (Clontech) and inserted in place of the GFP coding sequence of TGN38-EGFP. Nmnat2ΔPSΔBR-Nterex6-PA_GFP was created by PCR amplification of Nmnat2 exon 6 and insertion at the N-terminus of Nmnat2ΔPSΔBR-PA_GFP. The N-terminal TGN38 targeting sequence was made by fusing the TGN38 signal peptide sequence (a.a. 1–20) to its transmembrane domain surrounded by linker sequences (a.a. 281–330). This sequence was then inserted at the N-termini of Nmnat2ΔPSΔBR and Nmnat2Δex6 to create Nmnat2ΔPSΔBR-NterTGN and Nmnat2Δex6-NterTGN, respectively. Nmnat2ΔPSΔBR-NterMOM was created by addition of amino acids 1–37 of *Mus musculus* TOM20 to the N-terminus of Nmnat2ΔPSΔBR. Nmnat2Δex6H24D was created by site-directed mutagenesis of Nmnat2Δex6.

For organelle markers, the following accession numbers were used for PCR primer design. Constructs were amplified from mouse brain cDNA and inserted into the MCS of pEGFP-NI (Clontech) or ptagRFP-NI (Evrogen) vectors. TGN38-EGFP, NM_009443; Syntaxin6-EGFP, NM_021433; Synaptophysin-EGFP, NM_009305; mito-tagRFP, AK003116 (bp 1–72); LmanI-EGFP, AK011495; Golga2-EGFP, NM_133852; GAP43-EGFP, BC080758; SynaptotagminI-EGFP, NM_001252341. All constructs were verified by DNA sequencing (Beckman Coulter Genomics).

Nmnat2 deletion mutants (Δ32, Δ43, and Δ69) were a gift from Prof. Giulio Magni (Ancona, Italy). GST-Dsk2 UBA was kindly provided by Dr. Simon Cook (Cambridge, UK). The SNAP25-EGFP construct was a gift from Dr. Luke Chamberlain (Glasgow, UK). GFP-Bassoon was a gift from Prof. Eckart Gundelfinger (Magdeburg, Germany). Lamp1-RFP [53] was from Addgene (plasmid 1817).

### Animals

All animal work was carried out in accordance with the Animals (Scientific Procedures) Act, 1986, under Project License 80/2254. C57BL/6JOlaHsd mice were obtained from Harlan UK (Bicester, UK).

### Primary Neuronal Cell Culture

Dissociated superior cervical ganglia cultures were prepared and maintained in culture as described previously [13]. For live-cell imaging of axonal transport, cells were transferred (on the day of imaging) into imaging medium in order to improve performance and detectability of fluorescent proteins [54]. Cells were viable and appeared morphologically normal in imaging medium for at least 3 days. Imaging medium consisted of 1.80 mM CaCl_2_ (Sigma), 0.25 µM Fe(NO_3_)_3_ (Sigma), 0.81 mM MgSO_4_ (AnalaR), 5.33 mM KCl (AnalaR), 44.05 mM NaHCO_3_ (Sigma), 110.34 mM NaCl (AnalaR), 0.92 mM NaH_2_PO_4_ (Sigma), 4,500 mg/l glucose (AnalaR), 110 mg/l sodium pyruvate(PAA), 2 mM glutamine (Invitrogen), 100 ng/ml 7S NGF (Invitrogen), 1% penicillin/streptomycin (Invitrogen), 4 µM aphidicolin (Calbiochem), 1× MEM amino acids (PAA), 30 mg/l glycine (AnalaR), and 42 mg/l serine (Sigma) in sterile distilled water. For live-cell imaging of photoactivatable GFP (PA_GFP), cells were transferred (on the day of imaging) into Hibernate-E medium (Invitrogen) with added 2 mM glutamine (Invitrogen), 100 ng/ml 7S NGF (Invitrogen), 1% penicillin/streptomycin (Invitrogen), 2% B27 supplement (Invitrogen), and 4 µM aphidicolin (Calbiochem). For heterodimerisation, 500 nM A/C heterodimeriser (Clontech) was added 8 h before imaging. Where indicated, 40 µM 2-BP (Sigma) was added immediately after microinjection.

### Microinjection

DNA microinjections into the nuclei of primary culture SCG neurons were performed as described [13]. For dual-labelling live cell imaging, both DNA constructs were used at a concentration of 0.03 µg/µl in the injection mix. For single-color axonal transport imaging, constructs were used at 0.05 µg/µl in the injection mix. For photoactivation experiments, 0.05 µg/µl of the photoactivation construct was co-injected with 0.01 µg/µl mCherry expression construct except for experiments involving heterodimerisation where 0.03 µg/µl each of DmrA- and DmrC-tagged constructs were co-injected with 0.01 µg/µl mCherry expression construct. Seventy-five cells were injected per dish, and imaging was performed 24 h after microinjection. For experiments on neurite degeneration after cut, 0.01 µg/µl DsRed2 expression vector was co-injected with the relevant Nmnat2 constructs at varying concentrations (see text). We injected 75–100 cells in each dish and neurites were cut 48 h after microinjection. MitoTracker Red CMXRos (Invitrogen) was used according to the manufacturer's instructions.

### Live Imaging of Axonal Transport and Image Analysis

Time-lapse imaging of axonal transport was performed on an Olympus Cell^R^ imaging system (IX81 microscope, Hamamatsu ORCA ER camera, 100×1.45 NA apochromat objective, 485 and 561 nm laser excitation). During imaging, cell cultures were maintained at 37°C in an environment chamber (Solent Scientific). Images were captured at 4 (single-color) or 2.5 (dual-colour) frames per second for 1–2 min.

### Co-Migration Analysis

The extent of axonal co-migration of two fluorescent protein markers was analysed in time-lapse recordings of individual neurites. Using ImageJ software version 1.44 (Rasband, W.S., ImageJ, NIH, Bethesda, Maryland, USA, http://imagej.nih.gov/ij/, 1997–2011), the same neurite was straightened, contrast adjusted, and projected as a kymograph for both image stacks. The kymographs were then merged to create an overlay. Co-migration was scored for each particle trace according to whether it was detectable in only one or both of the kymograph channels. Co-migration was determined only for moving particles (i.e., traces that were not exclusively vertical in kymographs).

### Quantification of Axonal Transport Parameters

Parameters of axonal transport of fluorescently labelled proteins were determined from straightened time-lapse recordings of individual neurites using the Difference Tracker ImageJ software plugin [55]. Additionally, the percentage of moving and stationary particles was scored manually on kymographs.

### Photoactivation Imaging and Quantification

Photoactivation imaging was carried out on an Olympus FV1000 point scanning confocal microscope system (IX81 microscope, 60×1.35 NA plan super apochromat objective, 488 and 561 nm laser excitation). Microinjected cell bodies were identified based on their mCherry fluorescence. Imaging settings were adjusted to standard settings (5× zoom, scan rate 8 µm/s, frame rate 3 s/frame). After taking a pre-activation image, PA_GFP was activated by a 100 ms pulse of a 405 nm laser at 50% intensity in a 100 pixel region of interest in the cell body. Images were then taken every 3 s for a total of 5 min. For analysis, two circular regions of interest of identical size (50 pixel diameter) were selected in the cell body. One was placed in the originally activated area, while the other one was placed 10–20 µm away in an area that was not activated by the original laser pulse. For quantification, the percentage of combined fluorescence in these two areas that remained in the originally activated area was determined for each time point. Data were fitted for exponential decay, and decay constant (k) was calculated using GraphPad Prism 5.04.

### Quantification of Neurite Degeneration

Degeneration of ds-Red2 labelled neurites was determined for the same field of view at indicated time points after neurite transection. The percentage of neurites remaining continuous and morphologically normal compared to the initial time point was scored for each field. For experiments involving heterodimerisation, A/C heterodimeriser (Clontech) was added to the relevant dishes 8 h before cut at a final concentration of 500 nM. Fresh medium (with heterodimeriser where appropriate) was added 24 and 48 h after cut.

### HEK 293 Cell Culture and Transfection

HEK 293 cells were maintained in culture as described [13]. For transfection, cells were grown to 80% confluency in 10 cm or 24-well dishes and transfected using lipofectamine 2000 (Invitrogen) according to the manufacturer's instructions. For emetine chase experiments, HEK293 cells in 24-well dishes were co-transfected with Wld^S^-FLAG and the appropriate FLAG-Nmnat2 construct (turnover of Nmnat2 mutants) or with Flag-Nmnat1, TGN38-DmrC-HA, and DmrA-Nmnat2ΔPSΔBR-FLAG (turnover of re-targeted mutants). For relevant experiments, 100 µM 2-BP was added 6 h post-transfection. Twenty-four hours after transfection, cells were treated with 10 µM emetine (Sigma) and samples were taken at indicated time points.

For ubiquitination experiments, HEK293 cells in 10 cm dishes were transfected with the appropriate FLAG-Nmnat2 construct and, for re-targeting experiments, with empty pCMV-Tag4A or TGN38-DmrC-HA constructs. Twenty-four hours after transfection, 20 µM MG132 (Sigma) was added to the medium. Six hours later, cells were lysed and subjected to GST-Dsk2 UBA (wild-type or mutant) pulldown assay as described [38],[39]. Where indicated, 100 µM 2-BP was added 6 h post transfection.

### Western Blotting

SDS-PAGE analysis and Western blotting analysis were performed as described [13],[56]. Mouse monoclonal anti-FLAG (Sigma, M2) was used at 1∶3,000. AlexaFluor680-conjugated anti-mouse secondary antibody (Molecular Probes, Eugene, OR, USA) was used at 1∶5,000. Blots were scanned and quantified using the Odyssey imaging system (LI-COR Biosciences, Lincoln, NC, USA).

### Palmitate Labelling

HEK293 cells in six-well dishes were transfected with the appropriate FLAG-Nmnat2 construct. Twenty-four hours after transfection, 0.5 mCi/ml [9,10^3^H]-palmitate (Perkin Elmer) was added to the medium. After 6 h, cells were washed in PBS, lysed in 500 µl lysis buffer (20 mM Tris pH 7.5, 137 mM NaCl, 1 mM EGTA, 1% TritonX-100, 10% glycerol, 1.5 mM MgCl_2_, 50 mM NaF, 1 mM Na_3_VO_4_, and protease inhibitor mix (Roche); all chemicals AnalaR unless stated otherwise). The lysate was centrifuged for 10 min, 13,000 rpm. Following overnight incubation of the lysate with 5 µg of anti-FLAG antibody (Sigma), 50 µl of washed Sepharose beads (GE Healthcare) was added and mixed for another 3 h at 4°C. Beads were washed thrice in lysis buffer and twice in wash buffer (50 mM Tris, pH 8.0). Bound protein was eluted with Laemmli sample buffer (BioRad) and boiling for 5 min and processed for SDS-PAGE. After transfer to PVDF membrane, blots were dried and radiolabel was detected by exposure on Tritium phosphor screen (Fuji) for 14 d.

### Data Analysis

Statistical analyses and graph fitting were performed using GraphPad Prism 5.04 (GraphPad Software Inc.) and SPSS Statistics 19 (IBM).

## Supporting Information

Figure S1 Nmnat2 primary structure and mutants. (A) Primary structure of *Mus musculus* Nmnat2 with relevant regions highlighted: ISTID region (underlined), exon 6 (blue), C164/165 palmitoylation site (red, bold), and exon 6 basic residues (K151, K155, R162, R167, R172; orange). (B) Overview of Nmnat2 mutant constructs used in this study.
(TIF)

Figure S2 Palmitate labelling of Nmnat2 mutants. (A) Palmitate label and Western blot of wild-type and mutant Nmnat2. HEK293 cells expressing FLAG-Nmnat2 or one of its mutants were labelled with ^3^H palmitate, subjected to FLAG-immunoprecipitation, and processed for Phosphor Imaging and Western blot (see Materials and Methods for details). (B) Quantification of palmitate incorporation. Intensity of detected radiolabel was normalised to FLAG signal on Western blot for each construct. For presentation, mutant values were normalised to wild-type FLAG-Nmnat2. Error bars indicate SEM. *** indicates statistically significant difference between ΔPS and ΔBR mutants. (*** *p*<0.001).
(TIF)

Figure S3 Increased protection by untagged cytosolic Nmnat2 mutants. (A) Representative fields of view of primary culture SCG neurites 0 and 24 h after neurite cut, labelled by dsRed2 expression and injected with 0.01 µg/µl empty vector or the relevant untagged Nmnat2 variant. (B) Quantification of experiment in (A). Error bars indicate SEM. * indicates statistically significant difference compared to wild-type Nmnat2 (* *p*<0.05).
(TIF)

Figure S4 Untagged Nmnat2 deletion mutants are able to strongly preserve neurites. (A) Representative fields of view of distal primary culture SCG neurites 0 and 72 h after neurite cut, labelled by dsRed2 expression and injected with 0.0005 µg/µl empty vector or the relevant unlabelled Nmnat2 variant. (B) Quantification of experiment shown in (A). Error bars indicate SEM. *, **, and *** indicate statistically significant difference compared to wild-type Nmnat2 (* *p*<0.05, ** *p*<0.01, *** *p*<0.001).
(TIF)

Figure S5 Enzymatic activity is required for protection by Nmnat2Δex6. (A) Representative fields of view of distal primary culture SCG neurites 0 and 72 h after neurite cut, labelled by dsRed2 expression and injected with 0.0005 µg/µl Nmnat2Δex6-EGFP or enzyme-dead Nmnat2Δex6H24D. (B) Quantification of experiment shown in (A). Error bars indicate SEM. ** indicates statistically significant difference compared to Nmnat2Δex6 (** *p*<0.01).
(TIF)

Figure S6 2-Bromopalmitate treatment impairs Nmnat2 membrane targeting and extends Nmnat2 half-life. (A) Individual frames from photoactivation assay of SCG primary culture neurons expressing Nmnat2-PA_GFP in absence or presence of 40 µM 2-BP. (B) Quantification of protein mobility in (A). Error bars indicate SEM. *** indicates statistically significant difference compared to control (*** *p*<0.001). (C) Representative Western blot of GST-Dsk2 pulldown assay. HEK293 cells expressing FLAG-Nmnat2 in the presence or absence of 100 µM 2-BP. Cells were lysed (inp – total input) and ubiquitinated proteins were immunoprecipitated using GST-Dsk2 bound to glutathione beads (ubiq). Eluted proteins were processed for SDS-PAGE and analysed by Western blot using anti-FLAG antibody. (D) Quantification of ubiquitination assay in (C). For each condition, the total amount of ubiquitinated FLAG-Nmnat2 was normalised to total input. Error bars indicate SEM. * indicates statistically significant difference compared to control (* *p*<0.05). (E) Representative Western blot of HEK293 cells co-transfected with FLAG-Wld^S^ and FLAG-Nmnat2 in presence or absence of 100 µM 2-BP. Twenty-four hours after transfection, cells were treated with 10 µM emetine for the amount of time indicated, after which samples were processed for SDS-PAGE and Western blot using anti-FLAG antibody. (F) Quantification of Nmnat2 turnover after emetine treatment in (E). For each sample and time point the amount of FLAG-Nmnat2 remaining was normalised to FLAG-Wld^S^ as an internal control. Error bars indicate SEM. Half-life of Nmnat2 was significantly reduced by treatment with 2-BP (from 45 to 65 min, *p*<0.01).
(TIF)

Figure S7 Successful re-targeting to membranes of Nmnat2ΔPSΔBR mutant by N-terminal tags. (A) Individual frames from photoactivation assay of SCG primary culture neurons expressing Nmnat2ΔPSΔBR-PA_GFP or one of its N-terminally membrane targeted variants (Nmnat2ΔPSΔBR-Nterex6-PA_GFP, Nmnat2ΔPSΔBR-NterTGN-PA_GFP, and Nmnat2ΔPSΔBR-NterMOM-PA_GFP). The region of activation is indicated by an orange circle in each image. (B) Quantification of protein mobility in (A). Error bars indicate SEM. (C) Representative images of cell bodies of primary culture SCG neurons expressing Nmnat2ΔPSΔBR-NterMOM-PA_GFP. The whole cell body was subjected to a 405 nm laser pulse in order to activate the entire pool of PA_GFP to enable co-localization analysis. Cells were then stained with MitoTracker dye.
(TIF)

Figure S8 Successful membrane re-targeting of Nmnat2ΔPSΔBR mutant by heterodimerisation. (A) Photoactivation assay of SCG primary culture cell bodies co-expressing DmrA-Nmnat2ΔPSΔBR-PA_GFP and TGN38-DmrC-HA in the absence (control) or presence of 500 µM A/C heterodimeriser for 8 h before imaging. The region of activation is indicated by an orange circle in each image. (B) Quantification of protein mobility in (A). Error bars indicate SEM.
(TIF)

Figure S9 Confirmation of re-targeting to membranes of Nmnat2Δex6 by N-terminal TGN38 tag. (A) Individual frames from photoactivation assay of SCG primary culture neurons expressing Nmnat2Δex6-PA_GFP or Nmnat2Δex6-NterTGN-PA_GFP. The region of activation is indicated by an orange circle in each image. (B) Quantification of protein mobility in (A). Error bars indicate SEM.
(TIF)

## Abbreviations

2-BP: 2-bromopalmitate
EGFP: enhanced green fluorescent protein
ERGIC: ER–Golgi intermediate compartment
GAP-43: Growth Associated Protein 43
ISTID: isoform-specific targeting and interaction domain
NAD: nicotinamide adenine dinucleotide
Nmnat: Nicotinamide mononucleotide adenlylyltransferase
PA_GFP: photoactivatable green fluorescent protein
SCG: superior cervical ganglion
Wld^S^: slow Wallerian degeneration protein
