# Supplementary figures and images for: Subcellular Localization Determines the Stability and Axon Protective Capacity of Axon Survival Factor Nmnat2
Source: PLoS Biol. 2013 Apr 16;11(4):e1001539. doi: 10.1371/journal.pbio.1001539 (PMC3627647; doi:10.1371/journal.pbio.1001539)

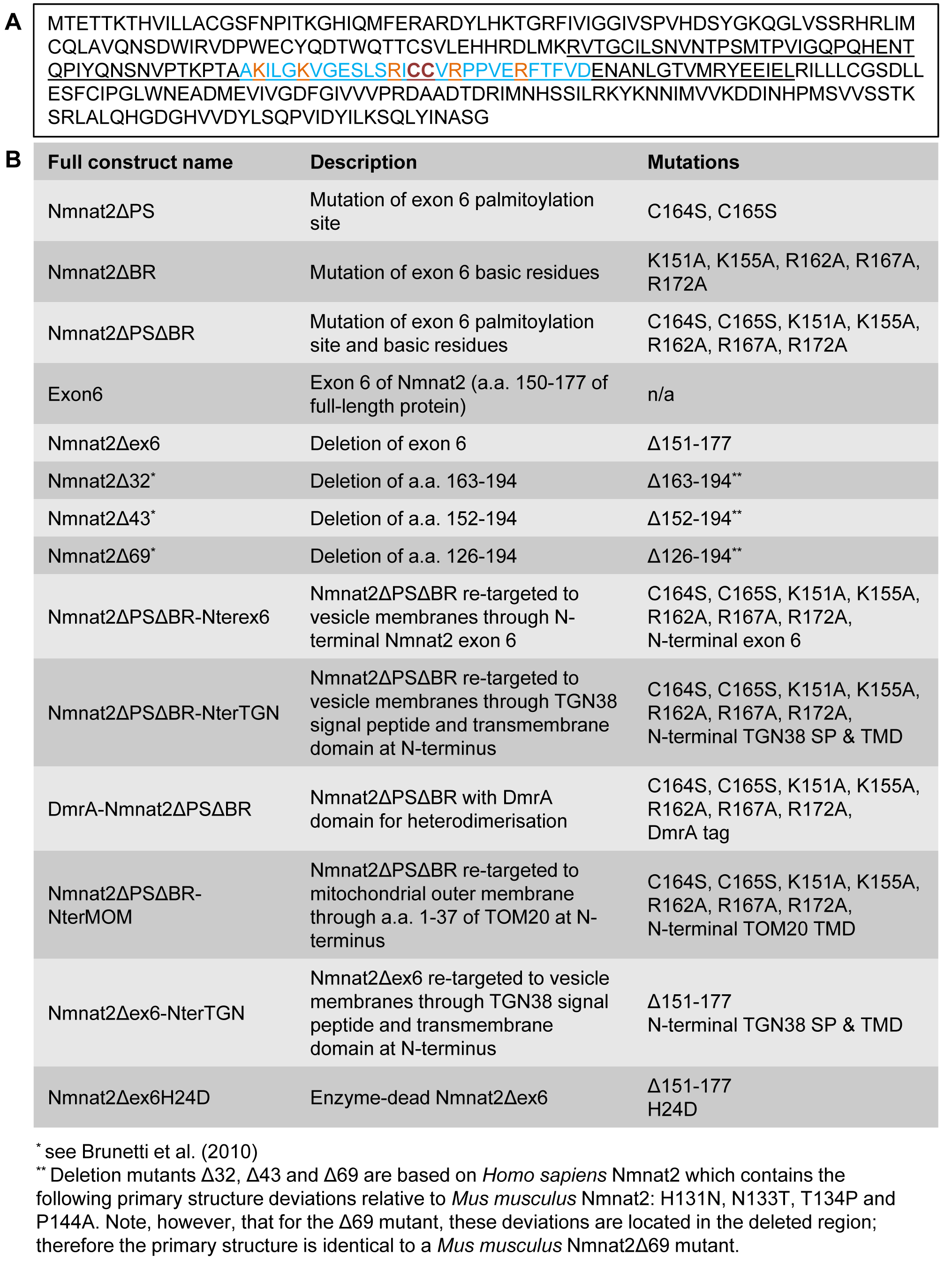

Supplement: Figure S1 — Nmnat2 primary structure and mutants. (A) Primary structure of Mus musculus Nmnat2 with relevant regions highlighted: ISTID region (underlined), exon 6 (blue), C164/165 palmitoylation site (red, bold), and exon 6 basic residues (K151, K155, R162, R167, R172; orange). (B) Overview of Nmnat2 mutant constructs used in this study. (TIF) [file pbio.1001539.s001.tif]

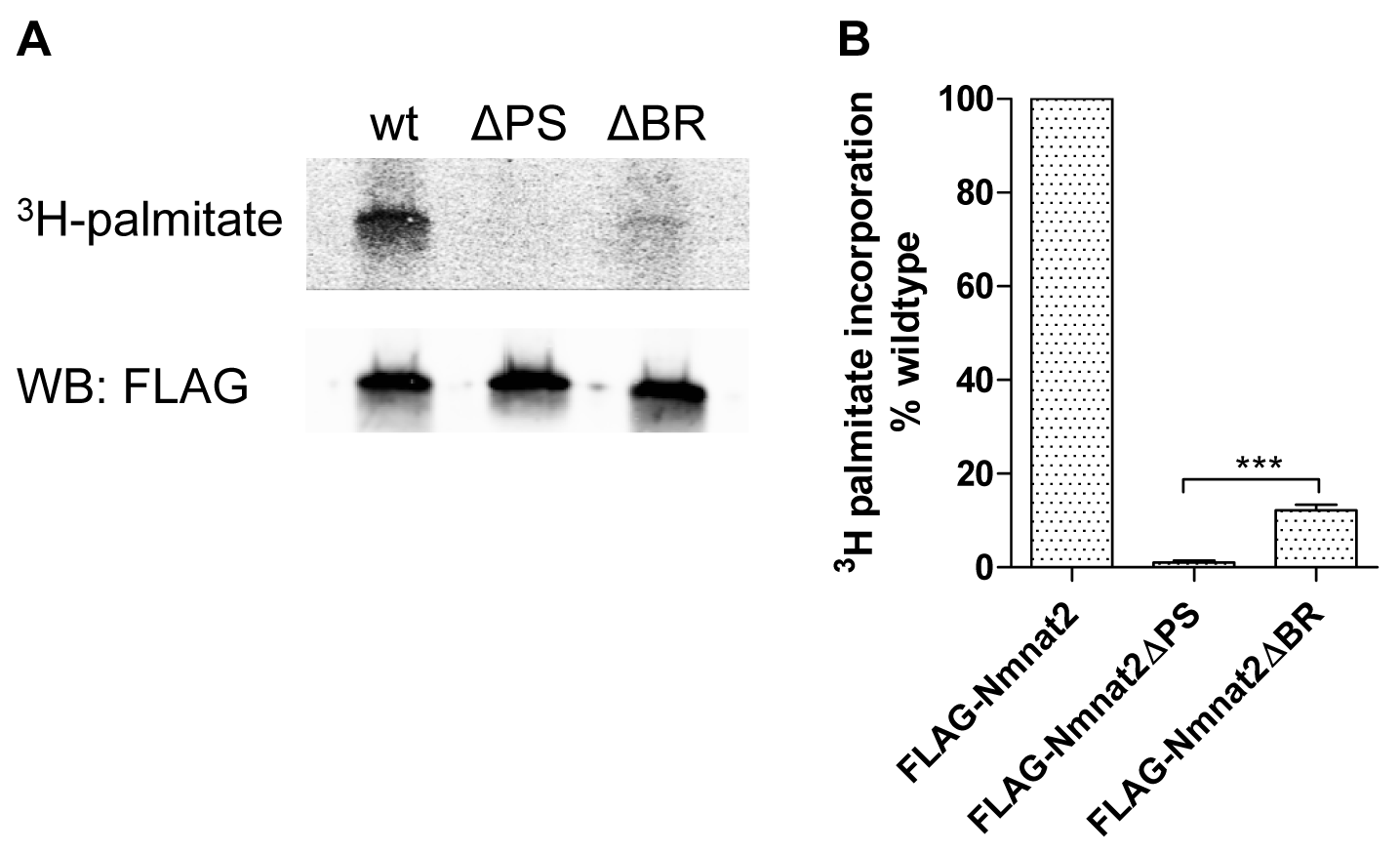

Supplement: Figure S2 — Palmitate labelling of Nmnat2 mutants. (A) Palmitate label and Western blot of wild-type and mutant Nmnat2. HEK293 cells expressing FLAG-Nmnat2 or one of its mutants were labelled with 3H palmitate, subjected to FLAG-immunoprecipitation, and processed for Phosphor Imaging and Western blot (see Materials and Methods for details). (B) Quantification of palmitate incorporation. Intensity of detected radiolabel was normalised to FLAG signal on Western blot for each construct. For presentation, mutant values were normalised to wild-type FLAG-Nmnat2. Error bars indicate SEM. *** indicates statistically significant difference between ΔPS and ΔBR mutants. (*** p<0.001). (TIF) [file pbio.1001539.s002.tif]

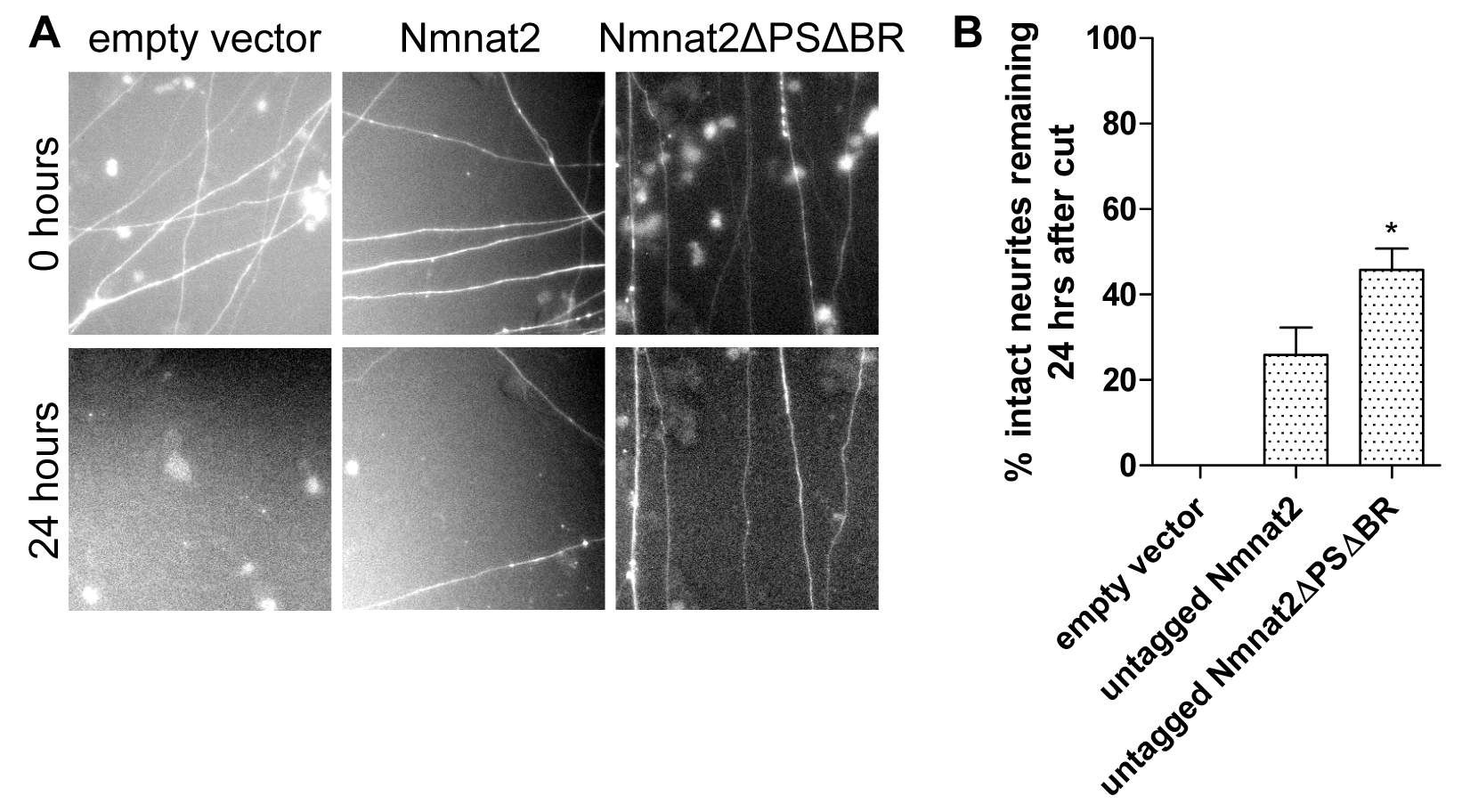

Supplement: Figure S3 — Increased protection by untagged cytosolic Nmnat2 mutants. (A) Representative fields of view of primary culture SCG neurites 0 and 24 h after neurite cut, labelled by dsRed2 expression and injected with 0.01 µg/µl empty vector or the relevant untagged Nmnat2 variant. (B) Quantification of experiment in (A). Error bars indicate SEM. * indicates statistically significant difference compared to wild-type Nmnat2 (* p<0.05). (TIF) [file pbio.1001539.s003.tif]

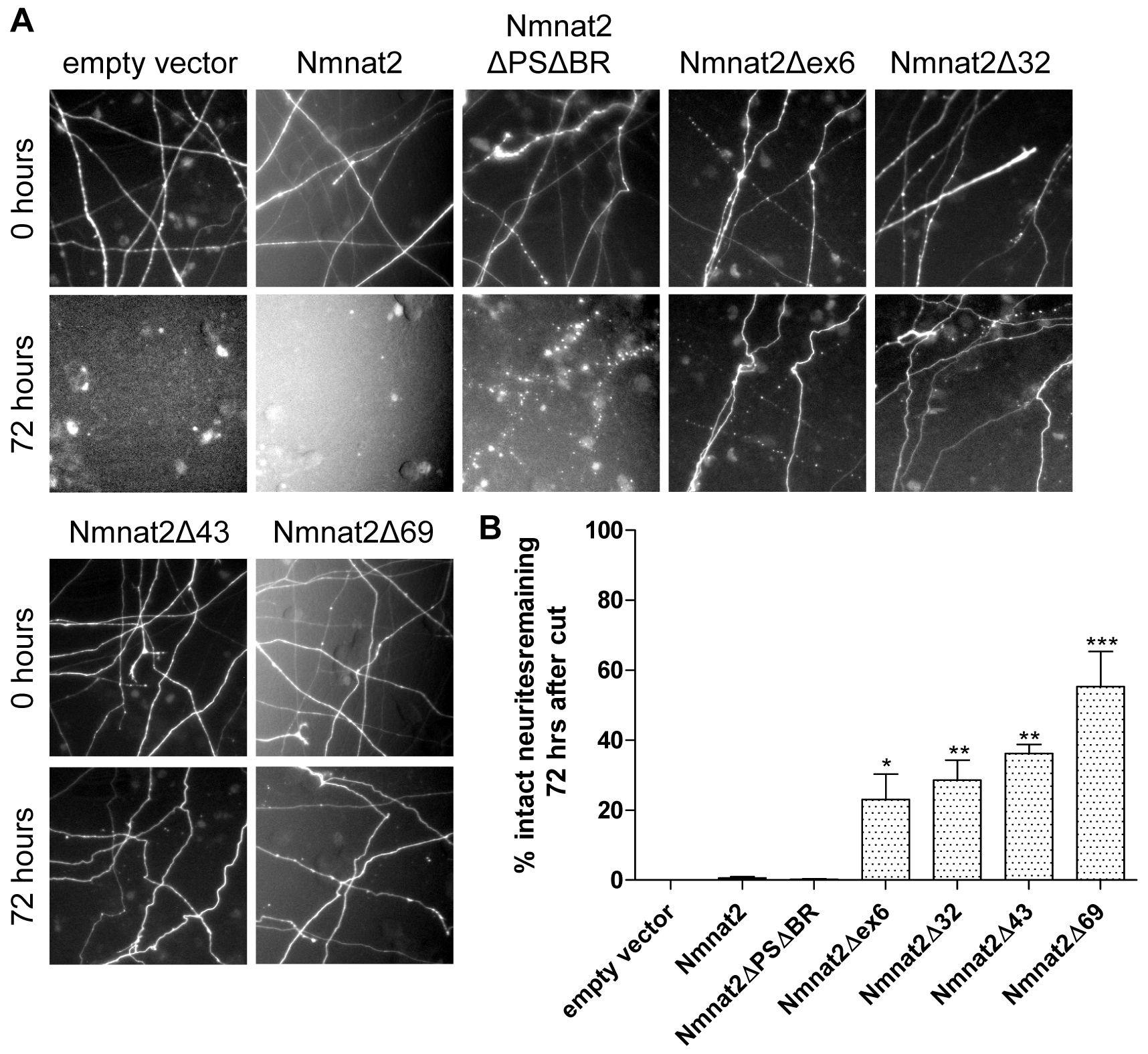

Supplement: Figure S4 — Untagged Nmnat2 deletion mutants are able to strongly preserve neurites. (A) Representative fields of view of distal primary culture SCG neurites 0 and 72 h after neurite cut, labelled by dsRed2 expression and injected with 0.0005 µg/µl empty vector or the relevant unlabelled Nmnat2 variant. (B) Quantification of experiment shown in (A). Error bars indicate SEM. *, **, and *** indicate statistically significant difference compared to wild-type Nmnat2 (* p<0.05, ** p<0.01, *** p<0.001). (TIF) [file pbio.1001539.s004.tif]

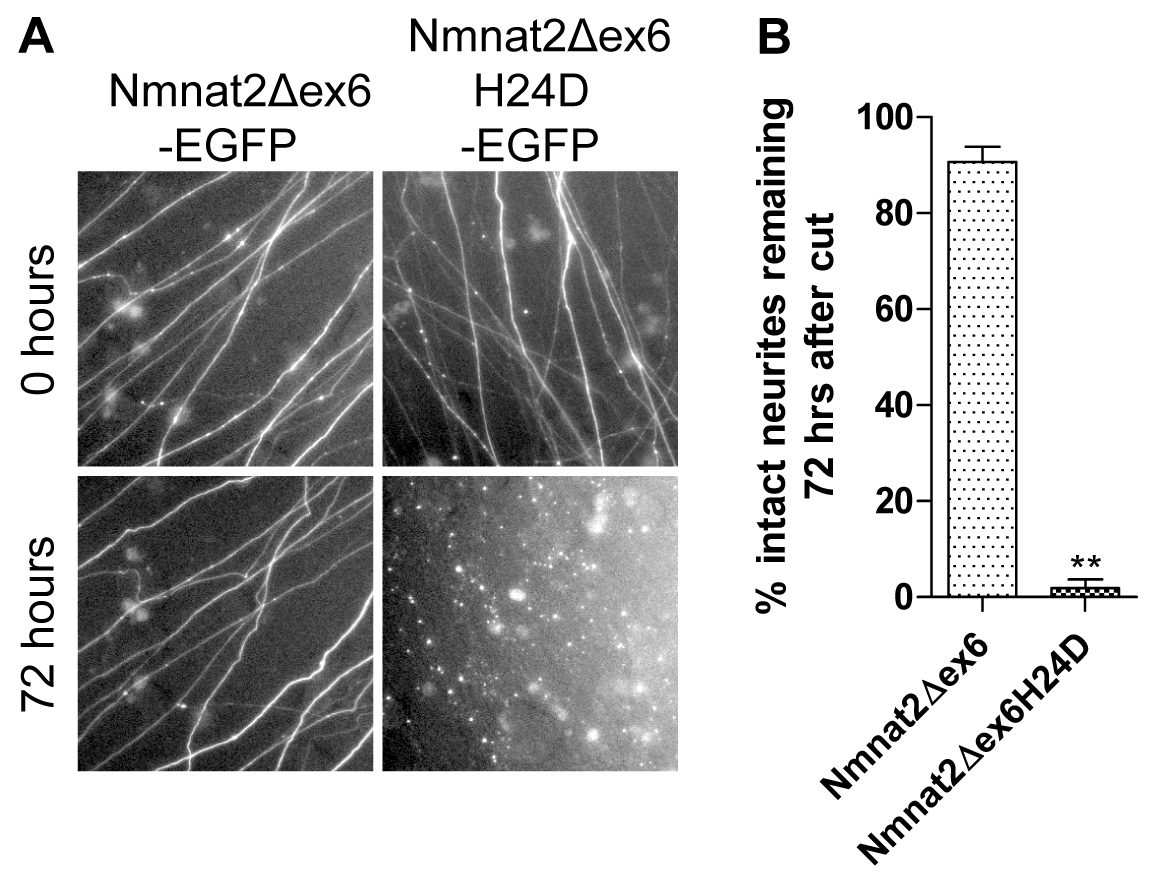

Supplement: Figure S5 — Enzymatic activity is required for protection by Nmnat2Δex6. (A) Representative fields of view of distal primary culture SCG neurites 0 and 72 h after neurite cut, labelled by dsRed2 expression and injected with 0.0005 µg/µl Nmnat2Δex6-EGFP or enzyme-dead Nmnat2Δex6H24D. (B) Quantification of experiment shown in (A). Error bars indicate SEM. ** indicates statistically significant difference compared to Nmnat2Δex6 (** p<0.01). (TIF) [file pbio.1001539.s005.tif]

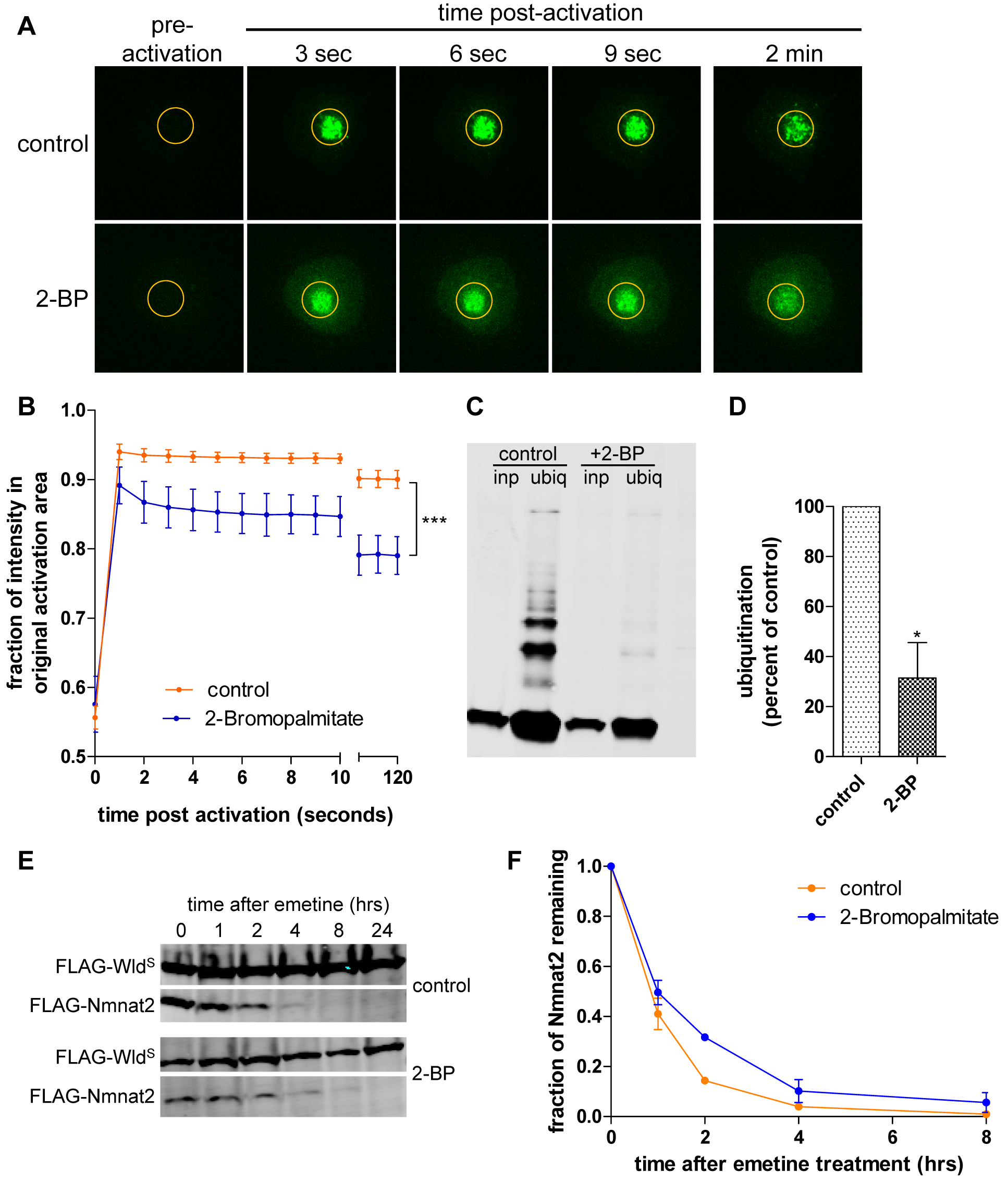

Supplement: Figure S6 — 2-Bromopalmitate treatment impairs Nmnat2 membrane targeting and extends Nmnat2 half-life. (A) Individual frames from photoactivation assay of SCG primary culture neurons expressing Nmnat2-PA_GFP in absence or presence of 40 µM 2-BP. (B) Quantification of protein mobility in (A). Error bars indicate SEM. *** indicates statistically significant difference compared to control (*** p<0.001). (C) Representative Western blot of GST-Dsk2 pulldown assay. HEK293 cells expressing FLAG-Nmnat2 in the presence or absence of 100 µM 2-BP. Cells were lysed (inp – total input) and ubiquitinated proteins were immunoprecipitated using GST-Dsk2 bound to glutathione beads (ubiq). Eluted proteins were processed for SDS-PAGE and analysed by Western blot using anti-FLAG antibody. (D) Quantification of ubiquitination assay in (C). For each condition, the total amount of ubiquitinated FLAG-Nmnat2 was normalised to total input. Error bars indicate SEM. * indicates statistically significant difference compared to control (* p<0.05). (E) Representative Western blot of HEK293 cells co-transfected with FLAG-WldS and FLAG-Nmnat2 in presence or absence of 100 µM 2-BP. Twenty-four hours after transfection, cells were treated with 10 µM emetine for the amount of time indicated, after which samples were processed for SDS-PAGE and Western blot using anti-FLAG antibody. (F) Quantification of Nmnat2 turnover after emetine treatment in (E). For each sample and time point the amount of FLAG-Nmnat2 remaining was normalised to FLAG-WldS as an internal control. Error bars indicate SEM. Half-life of Nmnat2 was significantly reduced by treatment with 2-BP (from 45 to 65 min, p<0.01). (TIF) [file pbio.1001539.s006.tif]

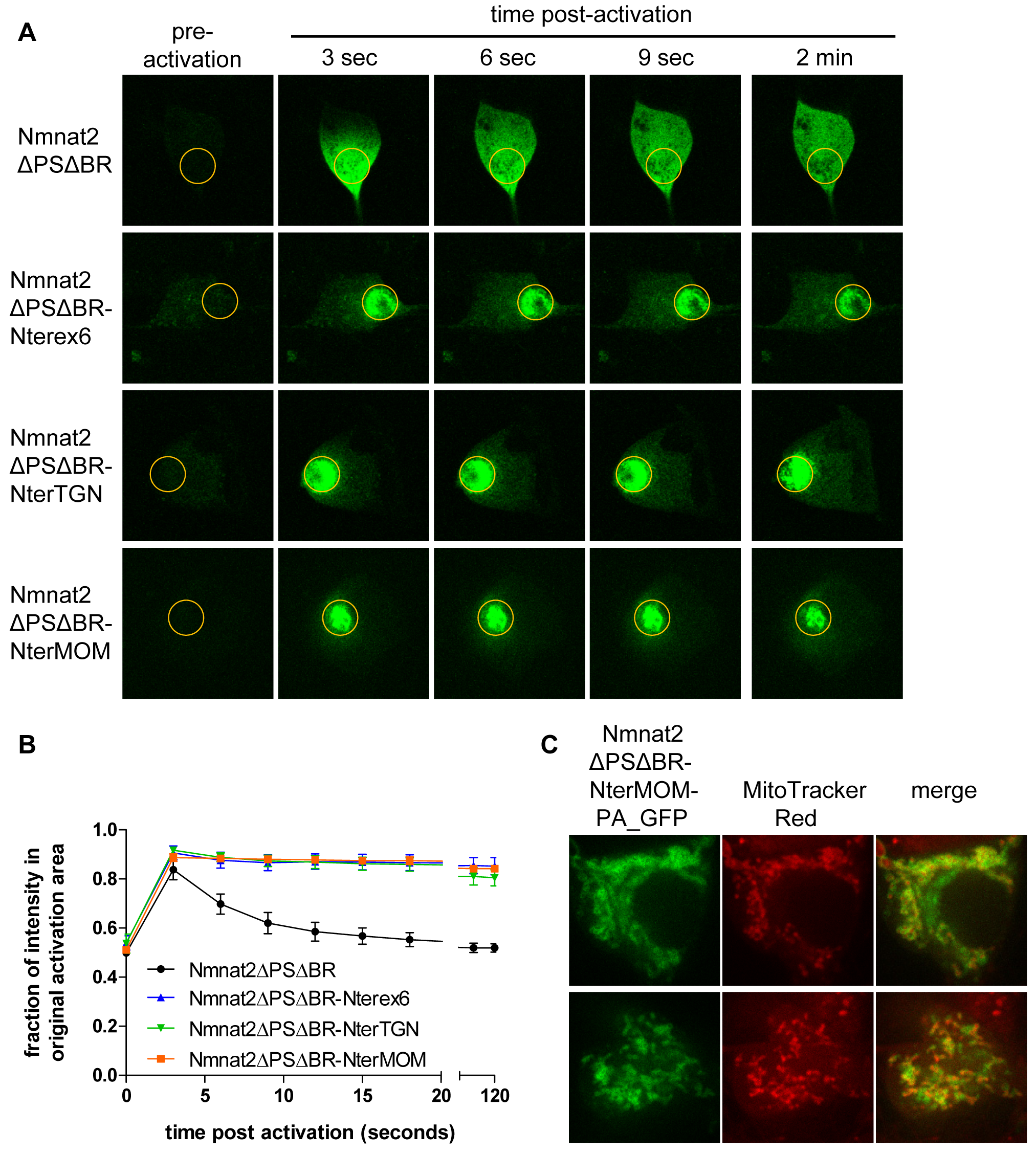

Supplement: Figure S7 — Successful re-targeting to membranes of Nmnat2ΔPSΔBR mutant by N-terminal tags. (A) Individual frames from photoactivation assay of SCG primary culture neurons expressing Nmnat2ΔPSΔBR-PA_GFP or one of its N-terminally membrane targeted variants (Nmnat2ΔPSΔBR-Nterex6-PA_GFP, Nmnat2ΔPSΔBR-NterTGN-PA_GFP, and Nmnat2ΔPSΔBR-NterMOM-PA_GFP). The region of activation is indicated by an orange circle in each image. (B) Quantification of protein mobility in (A). Error bars indicate SEM. (C) Representative images of cell bodies of primary culture SCG neurons expressing Nmnat2ΔPSΔBR-NterMOM-PA_GFP. The whole cell body was subjected to a 405 nm laser pulse in order to activate the entire pool of PA_GFP to enable co-localization analysis. Cells were then stained with MitoTracker dye. (TIF) [file pbio.1001539.s007.tif]

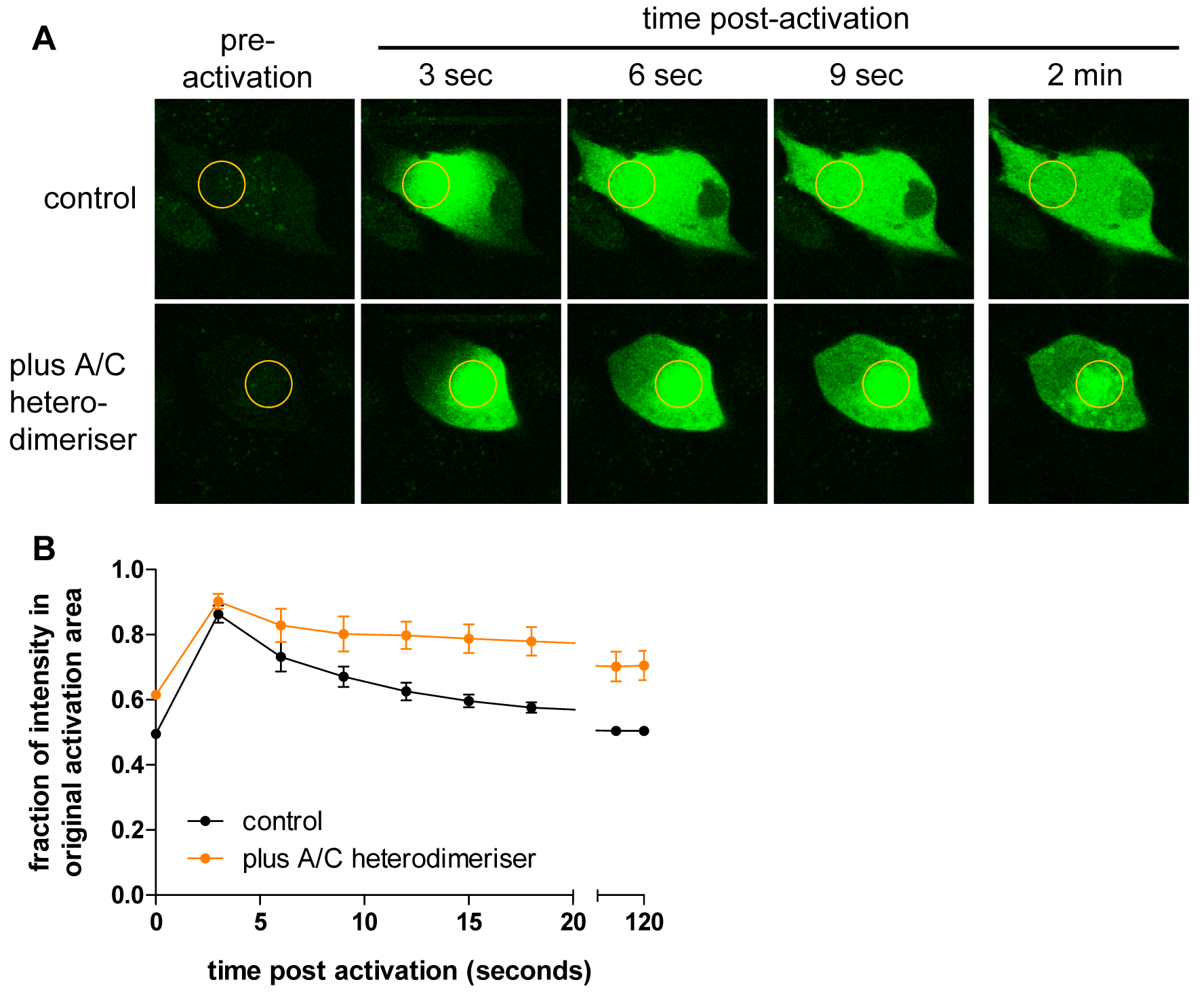

Supplement: Figure S8 — Successful membrane re-targeting of Nmnat2ΔPSΔBR mutant by heterodimerisation. (A) Photoactivation assay of SCG primary culture cell bodies co-expressing DmrA-Nmnat2ΔPSΔBR-PA_GFP and TGN38-DmrC-HA in the absence (control) or presence of 500 µM A/C heterodimeriser for 8 h before imaging. The region of activation is indicated by an orange circle in each image. (B) Quantification of protein mobility in (A). Error bars indicate SEM. (TIF) [file pbio.1001539.s008.tif]

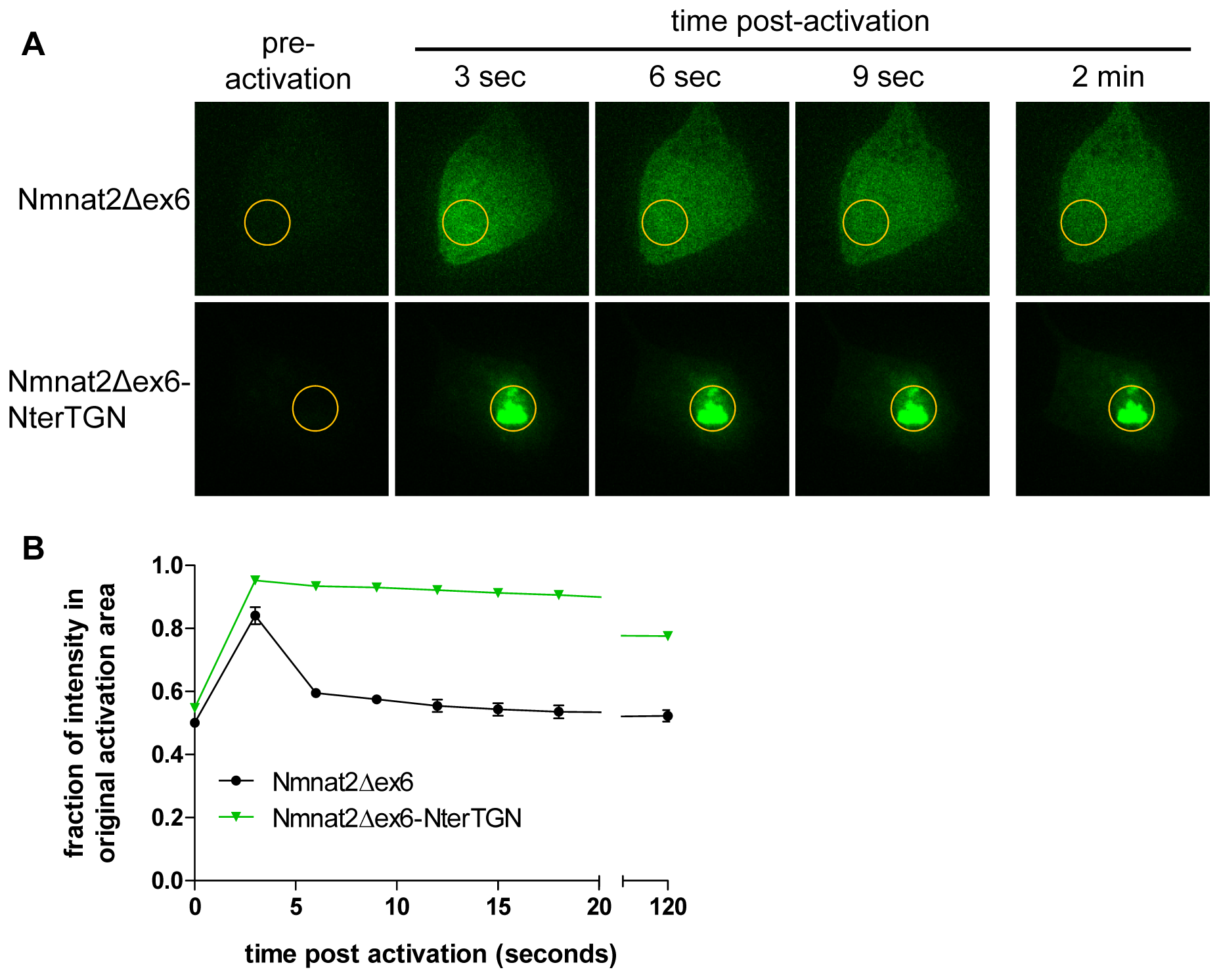

Supplement: Figure S9 — Confirmation of re-targeting to membranes of Nmnat2Δex6 by N-terminal TGN38 tag. (A) Individual frames from photoactivation assay of SCG primary culture neurons expressing Nmnat2Δex6-PA_GFP or Nmnat2Δex6-NterTGN-PA_GFP. The region of activation is indicated by an orange circle in each image. (B) Quantification of protein mobility in (A). Error bars indicate SEM. (TIF) [file pbio.1001539.s009.tif]
